# Structure-dependence and metal-dependence on atomically dispersed Ir catalysts for efficient n-butane dehydrogenation

**DOI:** 10.1038/s41467-023-38361-4

**Published:** 2023-05-05

**Authors:** Xiaowen Chen, Xuetao Qin, Yueyue Jiao, Mi Peng, Jiangyong Diao, Pengju Ren, Chengyu Li, Dequan Xiao, Xiaodong Wen, Zheng Jiang, Ning Wang, Xiangbin Cai, Hongyang Liu, Ding Ma

**Affiliations:** 1 Shenyang National Laboratory for Materials Science, Institute of Metal Research, Chinese Academy of Sciences, Shenyang, 110016 P. R. China; 2 School of Materials Science and Engineering, University of Science and Technology of China, Shenyang, 110016 P. R. China; 3 Beijing National Laboratory for Molecular Sciences, College of Chemistry and Molecular Engineering, Peking University, Beijing, 100871 P. R. China; 4 State Key Laboratory of Coal Conversion, Institute Coal Chemistry, Chinese Academy of Sciences, Taiyuan, 030001 P. R. China; 5 National Energy Center for Coal to Clean Fuel, Synfuels China Co., Ltd, Beijing, 100871 P. R. China; 6 The University of Chinese Academy of Sciences, Beijing, 100049 P.R. China; 7 Center for Integrative Materials Discovery, Department of Chemistry and Chemical and Biomedical Engineering, University of New Haven, West Haven, CT 06516 USA; 8 Shanghai Institute of Applied Physics, Chinese Academy of Sciences, Shanghai, 201204 P. R. China; 9 Department of Physics and Center for Quantum Materials, Hong Kong University of Science and Technology, Kowloon, Hong Kong SAR P. R. China

**Keywords:** Chemical engineering, Heterogeneous catalysis, Catalyst synthesis

## Abstract

Single-site pincer-ligated iridium complexes exhibit the ability for C-H activation in homogeneous catalysis. However, instability and difficulty in catalyst recycling are inherent disadvantages of the homogeneous catalyst, limiting its development. Here, we report an atomically dispersed Ir catalyst as the bridge between homogeneous and heterogeneous catalysis, which displays an outstanding catalytic performance for n-butane dehydrogenation, with a remarkable n-butane reaction rate (8.8 mol·g_Ir_^−1^·h^−1^) and high butene selectivity (95.6%) at low temperature (450 °C). Significantly, we correlate the BDH activity with the Ir species from nanoscale to sub-nanoscale, to reveal the nature of structure-dependence of catalyst. Moreover, we compare Ir single atoms with Pt single atoms and Pd single atoms for in-depth understanding the nature of metal-dependence at the atomic level. From experimental and theoretical calculations results, the isolated Ir site is suitable for both reactant adsorption/activation and product desorption. Its remarkable dehydrogenation capacity and moderate adsorption behavior are the key to the outstanding catalytic activity and selectivity.

## Abstract (web summary)

Single-site pincer-ligated iridium complexes exhibit the ability for C-H activation but suffer from instability and difficulty in catalyst recycling. Here the authors design an atomically dispersed Ir catalyst which can be considered the bridge between homogeneous and heterogeneous catalysis and displays an outstanding catalytic performance in n-butane dehydrogenation.

## Introduction

Alkenes are the irreplaceable cornerstone for value-added chemicals in the modern chemical industry. Catalytic dehydrogenation of alkanes from natural gas is a direct and simple method to produce alkenes. In fundamental research, alkane dehydrogenation can be conducted with either homogeneous or heterogeneous catalysts. The homogeneous catalysis includes transfer dehydrogenation and acceptorless dehydrogenation, which have made significant progress in the last three decades^1–4^. In 1979, Crabtree and co-workers pioneered the study of stoichiometric transfer dehydrogenation of alkanes, mediated by a homogeneous iridium pincer complex. With tert-butylethylene as a hydrogen acceptor, the single-site iridium pincer complex could catalyze the dehydrogenation of cyclooctane and cyclopentane and form iridium complexes of cyclooctadiene and cyclopentadiene^5^. In 1999, Goldman and co-workers reported the first catalytic system for regioselective transfer dehydrogenation of linear alkanes to α-olefins^6^, using pincer iridium complexes. Since then, various single-site pincer iridium complexes have been developed. Based on a (PCP)Ir coordination structure, the steric and electronic properties of pincer iridium complex can be precisely tuned by (1) substituting functional groups on two phosphorus atoms, (2) altering linkers between the backbone and two P atoms, (3) modifying para-position on the aromatic backbones or substituting other aromatic hydrocarbon backbones, and (4) introducing other metal atoms. The decreased steric hindrance in this type of catalyst favors catalytic activity and accelerates β-H elimination with regioselectivity to form terminal or internal olefin^7^. Moreover, strong linker-atom to C(aryl) π-donation can facilitate the rate-determining step in alkane C−H bond addition to 14e (pincer)Ir fragment, and thus enhance catalytic activity in the transfer dehydrogenation reaction^8^. However, limited by thermal stability, the highly efficient pincer iridium complexes suffered from decomposition under slightly harsh conditions (>300 °C). The difficulty of catalyst separation and recycling restricted large-scale industrial applications. To overcome the limitations, one solution is to immobilize iridium pincer complexes on oxide supports through covalent bonding to anchor Ir or para-position atoms. This strategy can be considered as the transformation of homogeneous catalysis to surface organometallic catalysis^2,9–11^. The highest stable temperature of single-site iridium pincer complexes on the supported oxides was above 300 °C, which was 100 °C higher than other homogeneous complexes with similar structures. But the collapse of the pincer ligand and loss of the Ir oxidation state caused fast and inevitable deactivation at >350 °C in heterogeneous alkane dehydrogenation^9^. Therefore, fabricating thermally stable isolated Ir atom catalysts to replace single-site iridium pincer complexes remains a challenge for alkane dehydrogenation.

For heterogeneous alkane dehydrogenation, C−H cleavage is active on noble metals such as Rh, Ru, Os, Ir, Pt, and Pd, which preferentially bind with −CH_3_, considered as carbon-preferred transition metals^12,13^. Previously, Ir nanoparticles (NPs) dispersed on oxide supports were designed for propane dehydrogenation (PDH). A second metal Sn was introduced as the promoter, which can separate larger Ir ensembles into highly dispersed and uniform Ir NPs and simultaneously modify the electronic property of the catalyst by forming IrSn alloy^14,15^. However, the Ir active species in the nanoscale has non-ideal atomic efficiency, resulting in poor activity in dehydrogenation reactions when normalized to Ir. In order to maximize the atomic efficiency, single-atom catalysts (SACs) have been designed for the alkane dehydrogenation process, such as nitrogen-doped carbon-supported Ru single atoms (SAs)^16^, γ-Al_2_O_3_ supported Pt_1_Cu single-atom alloy (SAA)^17^, SiO_2_ supported Rh_1_Cu SAA^18^, MFI siliceous zeolite confined single Fe sites^19^, SiO_2_ confined single Co sites^20^, and tetrahedral Co(II) sites^21^. For example, Zhang et al. designed the highly stable Ru SACs for PDH^16^. The Ru species remained almost unchanged compared with the fresh catalyst, even after reduction treatment by H_2_ at 600 °C or the PDH reactions, indicating that Ru species remained atomically dispersed and has partially oxidized state during PDH. Similarly, Jeffrey T. Miller et al. developed an γ-Al_2_O_3_ supported isolated Ni (II) site by anchoring Ni^2+^ cations into Al^3+^ vacancy on γ- Al_2_O_3_ as a catalyst for PDH^22^. The nature of the Ni sites remains constant for the fresh sample, regardless of 1 h treatment by 3% H_2_ at 600 °C or 3% C_3_H_8_ at 580 °C or 20% O_2_ at 600 °C, suggesting that the atomically dispersed Ni^2+^ sites retain the local structure over reduction or reaction-regeneration cycles. Benefiting from moderate strength in the adsorption of reactants and intermediates, SACs showed outstanding activity and desired selectivity in alkane dehydrogenation. Significantly, SACs with a simple geometric structure and coordination environment can serve as a model to provide fundamental insights into the reaction mechanism at the atomic level. Therefore, the highly active Ir SACs are still sought after in alkane dehydrogenation.

In this work, we fabricated a series of highly dispersed Ir catalysts on nanodiamond@graphene (ND@G), including atomically dispersed Ir atom (Ir_1_/ND@G), Ir sub-nanocluster (Ir_n_/ND@G) and Ir NPs (IrNPs/ND@G). Especially for the Ir_1_/ND@G catalyst, the Ir SAs were stabilized by the Ir-C bond on the ND@G surface, resulting in a similar structure with supported single-site iridium pincer complexes. In n-butane dehydrogenation (BDH), Ir_1_/ND@G showed a remarkable butane reaction rate (8.8 mol g_Ir_^−1^ h^−1^) with high butene selectivity (95.6%). Even at a relatively low temperature (450 °C), Ir_1_/ND@G showed a turnover frequency (TOF) of 0.48 s^−1^, 19.2 times higher than Ir_n_/ND@G and 34.3 times higher than IrNPs/ND@G. Significantly, we correlated the BDH activity with the average coordination numbers (CNs) of the Ir-Ir bond to reveal the structure-dependence from the nanoscale to the sub-nanoscale. We compared the Ir SAs with the catalysts of Pt SAs and Pd SAs to understand metal-dependence for BDH. Density functional theory (DFT) calculations results suggested that moderate adsorption of intermediates and easy desorption of butene on Ir SAs guaranteed high activity towards butene and remarkable catalyst stability.

## Results

### Preparation and characterization of highly dispersed Ir catalysts

**Fig. 1:**
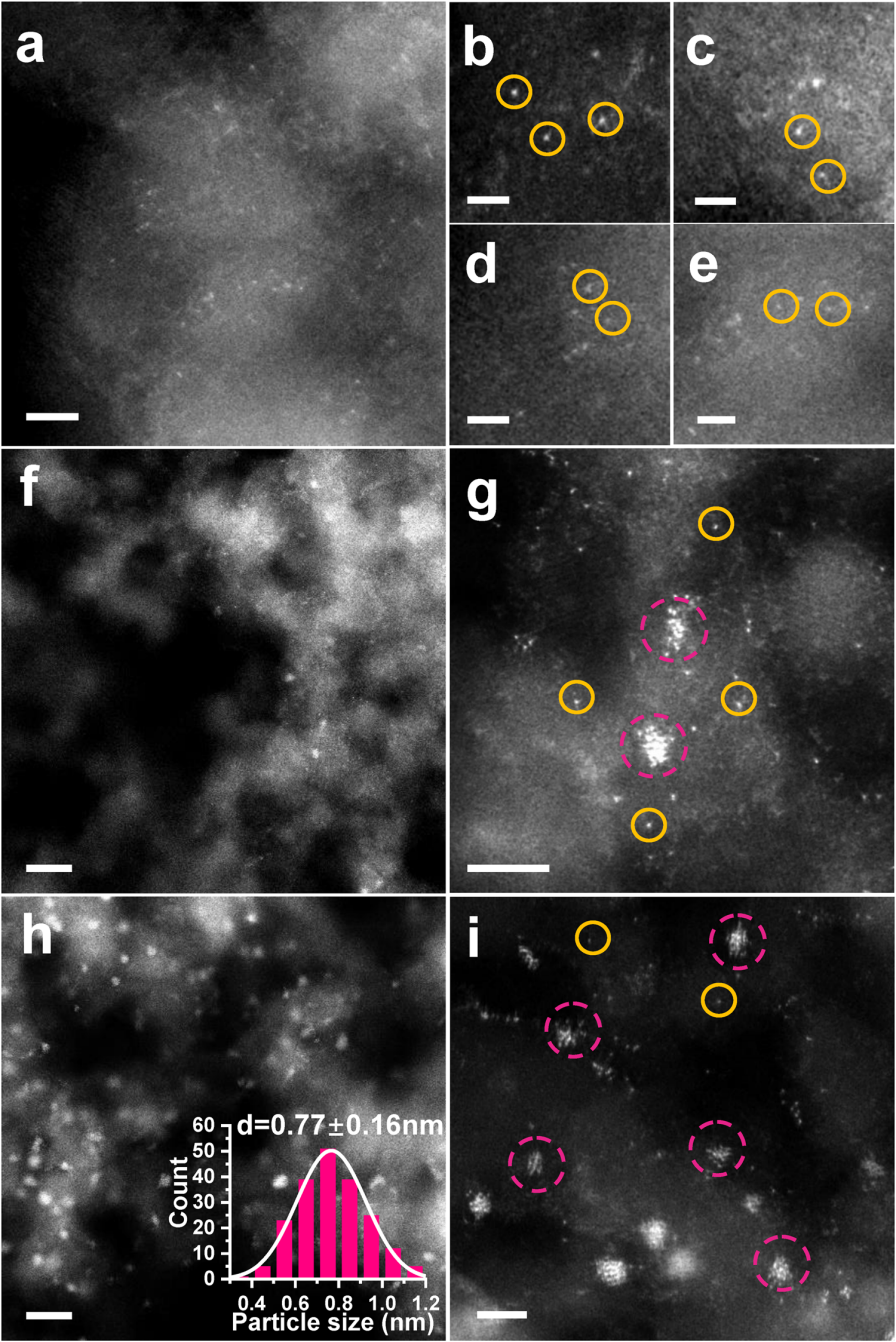
Microscopic characterization of Ir_1_/ND@G and Ir_1+n_/ND@G and Ir_n_/ND@G. **a**–**e** HAADF-STEM images of Ir_1_/ND@G. In the images, isolated Ir atoms are highlighted by yellow circles. **f**, **g** HAADF-STEM images of Ir_1+n_/ND@G. In the images, Ir clusters are highlighted by the pink circles. **h**, **i** HAADF-STEM images of Ir_n_/ND@G. Scale bars: **f**, **h**, 5 nm; **a**, **g**, **i**, 2 nm, and **b**–**e** 1 nm.

**Fig. 2:**
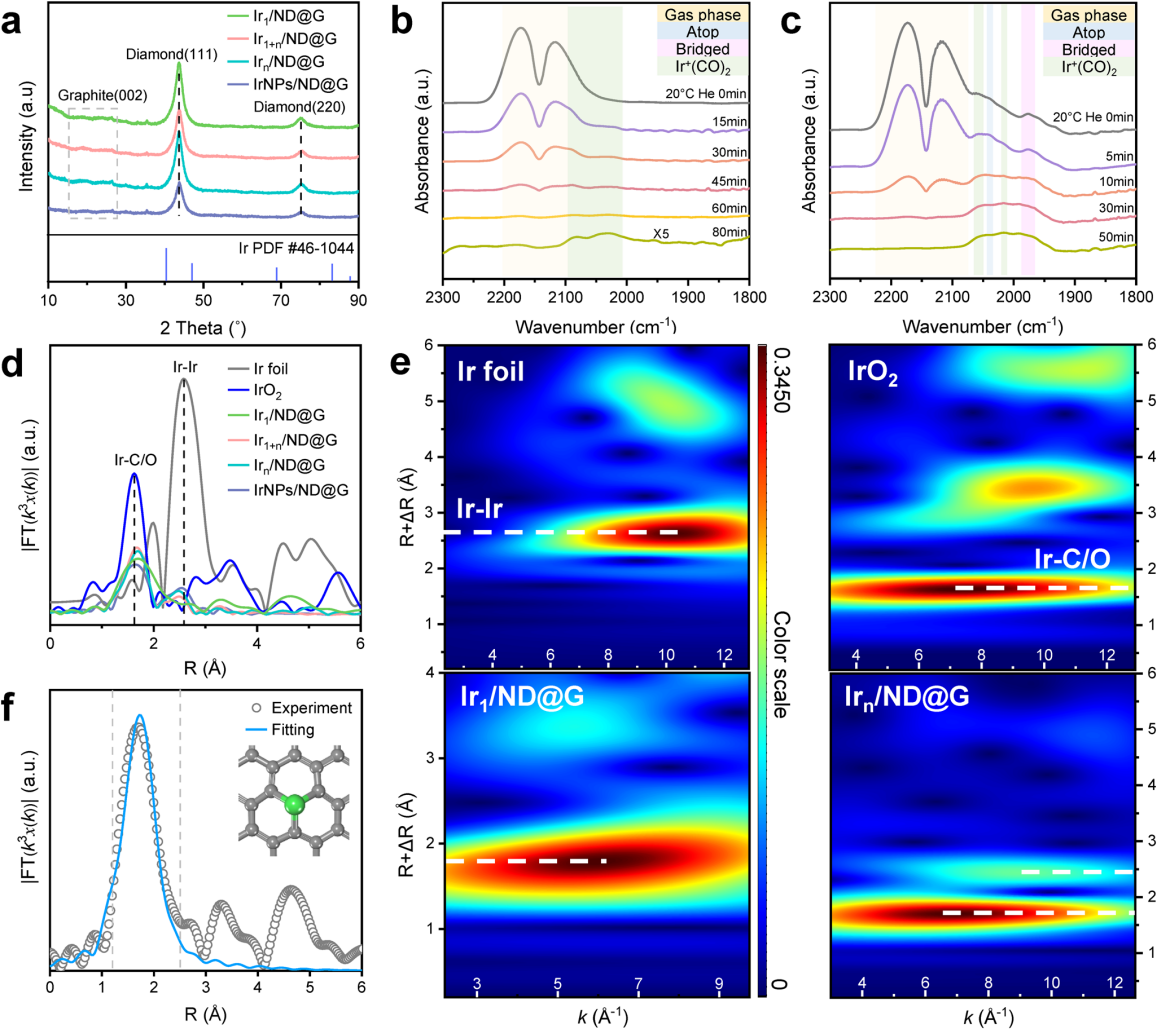
Structure characterizations of as-prepared catalysts. **a** XRD patterns of Ir_1_/ND@G, Ir_1+n_/ND@G, Ir_n_/ND@G, and IrNPs/ND@G. **b** In situ CO-DRIFTS of Ir_1_/ND@G. **c** In situ CO-DRIFTS of Ir_n_/ND@G. **d** Fourier-transformed *k*^3^-weighted EXAFS spectra of above as-prepared catalysts, Ir foil and IrO_2_. **e** WT analysis of Ir_1_/ND@G, Ir_n_/ND@G, Ir foil, and IrO_2_. **f** EXAFS fitting curve for Ir_1_/ND@G and the optimized Ir-C_3_ structure.

ND@G, composed of the *sp*^3^ diamond core and highly defective *sp*^2^ graphene outer shells, has been used as the support (Supplementary Fig. 1). The unique surface with abundant defects can trap and stabilize metal atoms by the metal–C bond. Its morphology and fine structure have been well studied in our previous reports^23–25^. A series of Ir/ND@G catalysts with different Ir loadings were prepared by the impregnation method or precipitation method, denoted as Ir_1_/ND@G (0.02 wt%), Ir_1+n_/ND@G (0.43 wt%), Ir_n_/ND@G (1.3 wt%), and IrNPs/ND@G (1.5 wt%), respectively. The physicochemical parameters of all the Ir/ND@G catalysts were summarized in Supplementary Table 1. To characterize the atomic-scale structure of catalysts, aberration-corrected high-angle annular dark-field scanning transmission electron microscopy (HAADF-STEM) was employed. For Ir_1_/ND@G, the Ir species was in the form of isolated Ir atoms without any Ir clusters and Ir NPs, as shown in Fig. 1a. The well-dispersed Ir atoms are highlighted by yellow circles in Fig. 1b–e. For Ir_1+n_/ND@G, the Ir clusters of a few atoms (highlighted by pink circles) began to appear among abundant isolated Ir atoms (Fig. 1f–g and Supplementary Fig. 2). As the Ir loading increased to 1.3 wt%, the island-like Ir clusters (*d* = 0.77 ± 0.16 nm, marked in pink circles) with 10–13 atoms (without crystalline structure) were clearly observed for Ir_n_/ND@G (Fig. 1h, i and Supplementary Fig. 3), indicating that the Ir species was well dispersed on the ND@G support. In contrast, in IrNPs/ND@G, the Ir NPs (nanoparticle diameter, *d* = 1.72 ± 0.30 nm) were located on the ND@G surface predominantly, together with a few isolated Ir atoms (Supplementary Fig. 4). The lattice spacing of Ir NPs was 0.22 nm, corresponding to the (111) facet of typical Ir NPs, implying the good crystallinity of the as-prepared Ir NPs on ND@G. Besides, from X-ray diffraction (XRD) profiles, the diffraction peaks were related to nanodiamond and graphite on the catalysts (Fig. 2a), indicating that the Ir species was highly dispersed on the ND@G surface, even for IrNPs/ND@G. These results are in good agreement with the HAADF-STEM observation.

In situ CO-diffuse reflectance infrared Fourier transform spectroscopy (CO-DRIFTS) is a powerful technology to study the surface structures of isolated Ir atoms, Ir clusters, and IrNPs in as-prepared catalysts. For Ir_1_/ND@G, a pair of CO adsorption peaks were apparently observed at 2086 and 2028 cm^−1^, respectively, which could be attributed to dicarbonyl species adsorbing on positively charged Ir species (Fig. 2b)^26–28^. The absence of other peaks suggested that no Ir multi-atomic species were present on ND@G. For Ir_n_/ND@G and IrNPs/ND@G, except for peaks of dicarbonyl species, two peaks appeared at 2050–2030 and 1980–1960 cm^−1^, respectively, which can be attributed to atop and bridged CO species adsorbing on metallic Ir species (Fig. 2c and Supplementary Fig. 5)^28,29^. Besides, for IrNPs/ND@G, a prominent peak of atop CO species demonstrated that large metallic Ir species have formed.

The Ir *L*_3_-edge X-ray absorption near-edge structure (XANES) spectroscopy and X-ray photoelectron spectroscopy (XPS) measurements revealed the average oxidation states of Ir on these samples. Generally, as decreasing the size of supported metal particles, the fraction of metal−support interface accordingly increased. Metal particles with higher electron-deficient charge states emerge from the strong charge transfer between the metal particles and the support, which results in the formation of positively charged metal species^30,31^. For Ir_1_/ND@G, the intensity of the white line was near IrO_2_, indicating the valence of Ir species was almost +4 valence^32,33^, which can be resulted from the atomic dispersion (Supplementary Fig. 6). When the Ir loading increases, the Ir 4*f*_7/2_ peak shifted towards lower binding energy, indicating that Ir species was gradually close to the metallic state along with the formation of Ir clusters or NPs (Supplementary Fig. 7)^34,35^. To further study the fine structure and local environment of Ir species, Fourier-transformed (FT) *k*^3^-weighted extended X-ray absorption fine structure (EXAFS) profiles were obtained (Fig. 2d). For Ir_1_/ND@G, the Ir-C/O scattering at ~1.6 Å was detected and no Ir-Ir scattering was observed, confirming that Ir species were atomically dispersed on ND@G. For Ir_1+n_/ND@G, Ir_n_/ND@G and IrNPs/ND@G, a distinct Ir-Ir scattering at ~2.6 Å was detected, and the average CN of Ir-Ir is 2.6, 3.5, and 3.6, respectively (Supplementary Table 2). Combined with the HAADF-STEM images, these results demonstrated the catalyst structure evolution from isolated Ir atoms to Ir clusters and then to Ir NPs, with the increase of Ir loading. The wavelet transformation (WT) of Ir *L*_3_-edge EXAFS oscillations visibly displayed the different forms of Ir species in both the *k* and R spaces. As shown in Fig. 2e, a maximum in the WT plot was observed at near 1.6 Å, which corresponds to the Ir-C/O back-scattering in Ir_1_/ND@G and Ir_n_/ND@G. Moreover, another weak peak emerged at near 2.6 Å in Ir_n_/ND@G, which was attributed to the Ir-Ir scattering, verifying the presence of Ir clusters with low CN. Quantitative chemical configuration analysis of Ir catalysts was carried out through the least-squared EXAFS fitting. The R space and *k* space fitting results are shown in Fig. 2f and Supplementary Figs. 8, 9. The corresponding structure parameters are listed in Supplementary Table 2. Based on these results, the proposed local atomic structure of Ir was constructed (see Fig. 2f). The isolated Ir atom was anchored over the defective sites of graphene by coordinating with three C atoms.

### Structure-dependence of Ir catalyst in alkane dehydrogenation

**Fig. 3:**
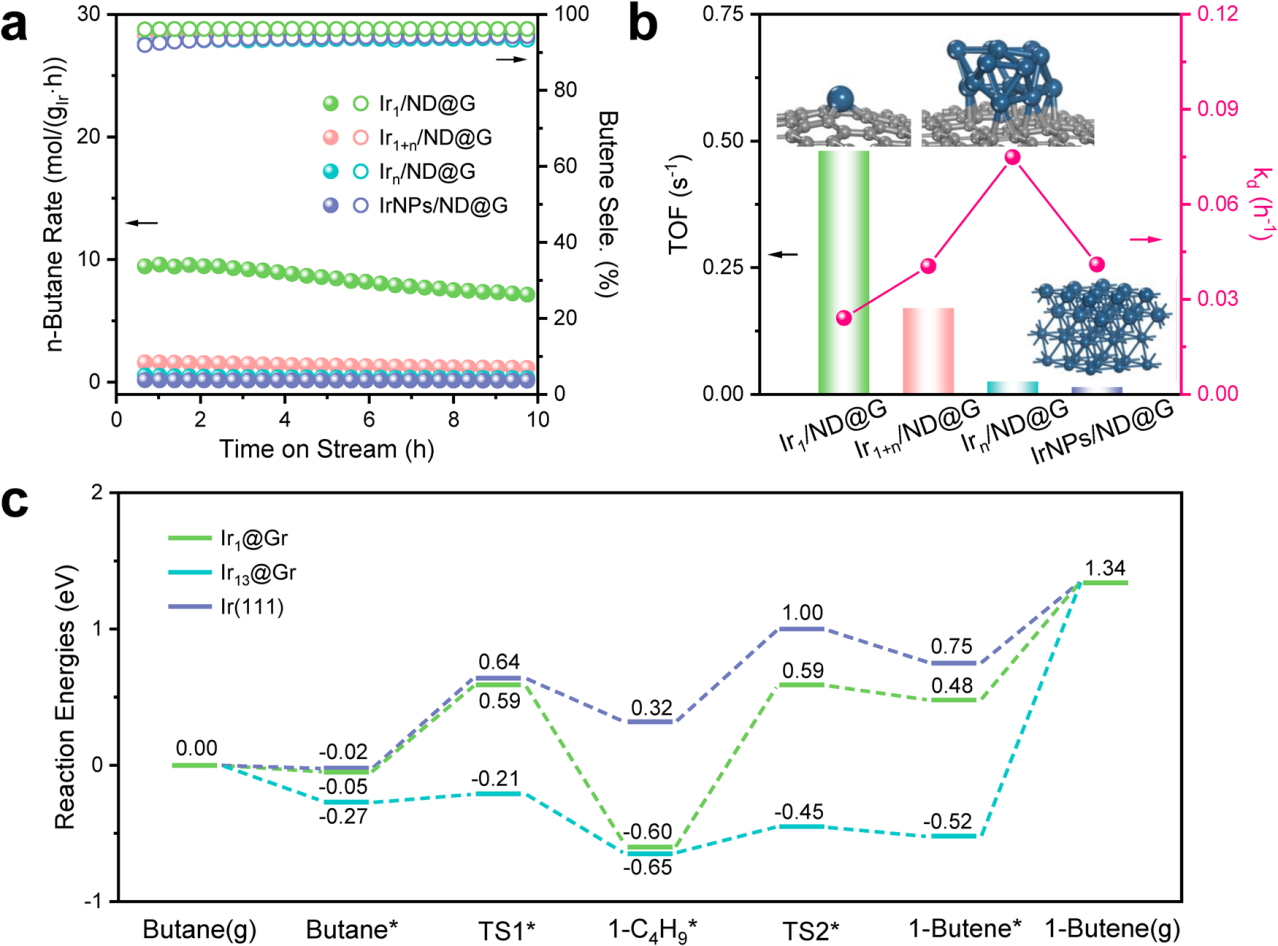
Catalytic performance for BDH. **a** n-Butane reaction rate and butene selectivity of Ir_1_/ND@G, Ir_1+n_/ND@G, Ir_n_/ND@G, and IrNPs/ND@G. **b** The value of TOF and *k*_d_. **c** Energy profiles of BDH on Ir_1_@Gr, Ir_13_@Gr, and Ir(111).

To gain insight into the structure-dependence of catalysts for alkane dehydrogenation, the catalytic performance of different Ir catalysts was evaluated for BDH (Fig. 3a and Supplementary Table 3). For Ir_n_/ND@G, the n-butane initial conversion was 19.2%, and the selectivity towards butene was 93.9% (Supplementary Fig. 10). The n-butane conversion dropped to 12.5%, and the value of *k*_d_ (deactivation rate constant) was 0.0749 h^−1^ in 10 h test (Fig. 3b). The stable structure on used Ir_n_/ND@G suggested the rapid deactivation resulting from coke formation to block Ir active sites (Supplementary Fig. 11 and Supplementary Table 4). When the Ir loading decreased, the reaction rate and butene selectivity increased simultaneously (Fig. 3a). For Ir_1+n_/ND@G, the reaction rate of n-butane (1.6 mol·g_Ir_^−1^·h^−1^) was 2.7 times higher than that of Ir_n_/ND@G (0.59 mol·g_Ir_^−1^·h^−1^) (Fig. 3a). Moreover, *k*_d_ decreased to 0.0405 h^−1^(Fig. 3b). The enhanced activity and stability indicated that the Ir-Ir CN might be the key factor for BDH. The structure of used Ir_1+n_/ND@G has been shown in Supplementary Fig. 11. Significantly, Ir_1_/ND@G with only 0.02 wt% Ir loading showed the highest activity and selectivity to butene (Fig. 3a). The reaction rate of n-butane on Ir_1_/ND@G reached 8.8 mol·g_Ir_^−1^·h^−1^ and remained 7.1 mol·g_Ir_^−1^·h^−1^ after 10 h, which was 5.5 times higher than that of Ir_1+n_/ND@G and 14.9 times higher than that of Ir_n_/ND@G. Moreover, the butene selectivity was as high as 95% throughout the process. To compare the butene selectivity at higher butane conversion, the mass of the Ir_1_/ND@G catalyst has been increased (Supplementary Fig. 12 and Supplementary Table 5). The Ir_1_/ND@G showed the highest butene selectivity, suggesting the undesired side reaction was well-restrained in the absence of Ir-Ir bonds. On the Ir_1_/ND@G, the n-butane initial conversion increased proportionally, confirming all the Ir atoms could directly participate in the reaction processes, including the adsorption and transformation of reactants. Moreover, the catalyst stability test was carried out for Ir_1_/ND@G by a 20-h and 50-h run (Supplementary Figs. 13,14). The n-butane reaction rate remained constant at 6.0 mol·g_Ir_^−1^·h^−1^ after 20 h. In long-term stability test for 50 h, the initial conversion of n-butane was 6.8% and still exhibited a considerable conversion of 4.1% after the 50 h reaction. The selectivity toward butene was as high as 95.7% after a 50 h reaction, and the value for *k*_d_ was only 0.0107 h^−1^. In contrast, IrNPs/ND@G, with few active Ir clusters and Ir SAs, showed delayed catalytic activity and selectivity (Supplementary Fig. 10).

Figure 3b illustrates the correlation between catalytic activity (or stability) and Ir species structure. The TOF increased with the structural evolution of Ir species from the nanoscale to the sub-nanoscale. The Ir_1_/ND@G with maximized atomic utilization showed a much higher n-butane reaction rate and TOF compared with the other catalysts with Ir ensemble sites, indicating that isolated Ir atoms were adequate for adsorbing n-butane and activating C-H bonds. Coke formation is a non-negligible cause for catalyst deactivation during BDH. Notably, the reduced stability (increased *k*_d_ value) was accompanied by the increasing size of Ir species. On Ir_n_/ND@G, the *k*_d_ value reached a maximum, indicating the atomically dispersed structure can suppress coke formation and retain outstanding catalytic activity during BDH. The amount of coke deposition on each used catalyst was measured by the thermogravimetric analysis (TGA) techniques, which was related to the size of Ir species on supported Ir catalysts (Supplementary Table 4). Additionally, the peak intensity ratio of D1 and G bands (*I*_D1_/*I*_G_) from Raman spectra is an important index to reflect the degree of graphitization on carbon materials. From the comparison of *I*_D1_/*I*_G_ between the fresh and used catalysts, lowered *I*_D1_/*I*_G_ on used catalysts suggests that increasing graphitic coke deposited during dehydrogenation (Supplementary Fig. 15)^17,36,37^. Besides, the Ir/ND@G samples were characterized after the BDH tests by STEM and XRD. After 10-h BDH, no sintering of Ir clusters or isolated Ir atoms into larger NPs was observed in any of the samples (Supplementary Figs. 11,16). After 0.5 h BDH, Ir species of Ir_1_/ND@G was atomically dispersed, indicating isolated Ir sites were the real active sites during the reaction process (Supplementary Fig. 17). The STEM images of Ir_1_/ND@G after 10 and 20 h BDH showed that few isolated Ir atoms was aggregated into small clusters, which attenuated the remarkable activity of isolated Ir atoms (Supplementary Figs. 16, 18). As a result, structural evolution from SAs to clusters was a crucial factor for Ir sub-nanoscale catalytic deactivation. In addition, the reusability of Ir_1_/ND@G catalyst has been also examined (Supplementary Fig. 19). The n-butane conversion and butene selectivity have been almost restored in two regeneration cycles. The *k*_d_ values of the two regeneration cycles were 0.0228 h^−1^and 0.0216 h^–1^, respectively. The butene selectivity was above 94% throughout the reusability test. In consideration of the stability of Ir_1_/ND@G at the initial reaction step, in situ CO-DRIFTS has been used to accurately identify the structure of Ir_1_/ND@G under reaction conditions and reduction conditions. For Ir_1_/ND@G under reaction condition or reduction treatment, a pair of CO adsorption peaks were apparently observed at 2100–2086 cm^−1^ and 2047–2017 cm^−1^, respectively, which could be attributed to dicarbonyl species adsorbing on positively charged Ir species (Supplementary Figs. 20,21). These results are consistent with those on the fresh sample (Fig. 2b). No peaks of atop and bridged CO species on Ir^0^ multi-atomic species have been observed, demonstrating isolated Ir atom bonded with C atoms was stable and reserved its positive valence at the initial period of the reaction. Therefore, we concluded that the Ir_1_/ND@G catalyst exhibited decent activity, high C_4_ olefins (or butene) selectivity and stability at relatively low temperatures, compared with the previously reported supported metal catalysts as displayed in Supplementary Table 6.

As shown in Fig. 3c, BDH into butene on model Ir_1_@Gr, Ir_13_@Gr, or Ir(111) surface (structural details in Supplementary Fig. 22) was computed by DFT to understand the activity difference induced by catalytic structure. Physical adsorption of butane was found on Ir(111) and Ir_1_@Gr, which was indicated by their adsorption energies (−0.02 and −0.05 eV, respectively). But the stronger adsorption of butane occurred on Ir_13_@Gr with the adsorption energies of −0.27 eV. Ir_13_@Gr exhibits much higher dehydrogenation activity than Ir(111) and Ir_1_@Gr, because of the strong adsorption of butane* (−0.27 eV), extremely low stepwise barrier (0.06 and 0.20 eV), and overall exothermicity (−0.52 eV) until the formation of 1-butene*. Different from Ir_13_@Gr, butane dehydrogenation into 1-butene* is endothermic by 0.48 eV on Ir_1_@Gr with an apparent barrier of 0.59 eV and is endothermic by 0.75 eV on Ir(111) with an apparent barrier of 1.00 eV. Therefore, the order of dehydrogenation activity is Ir_13_@Gr > Ir_1_@Gr > Ir(111). However, the difficult desorption of 1-butene* (1.86 eV) and overly superior dehydrogenation ability on Ir_13_@Gr expanded the possibility of over-dehydrogenation of 1-butene* and coke formation. Corrected Gibbs free energy profiles are shown in Supplementary Fig. 23. As a result, Ir_1_@Gr showed high catalytic performance with moderate catalytic activity and intermediate stability. Moreover, it should be noticed that the rate-determining step is significantly dependent on Ir species structure. On Ir_1_@Gr and Ir(111), the steps of C-H activation play primary roles, determining the dehydrogenation activity during the overall process. On Ir_13_@Gr, the strong adsorption of reaction intermediates makes C-H activation kinetically easy and further shifts the rate-determining step from C-H activation to butene desorption.

### Metal-dependence of noble metal catalysts in alkane dehydrogenation

Highly efficient Pt and Pd catalysts have been exploited for dehydrogenation reactions (such as alkane dehydrogenation and cycloalkane dehydrogenation) according to the higher activity towards C−H bond cleavage against C−C bond cleavage^25,38–42^. To disclose the cause for the spectacular activity over Ir_1_/ND@G, Pt_1_/ND@G, and Pd_1_/ND@G were prepared by the impregnation method, and the nature of metal-dependence was investigated for the BDH reaction. For Pt_1_/ND@G and Pd_1_/ND@G, the structure and morphology had been described in our previous reports^23,25,43^. All the Pd atoms and Pt atoms were atomically dispersed on ND@G support without visible NPs or clusters.

**Fig. 4:**
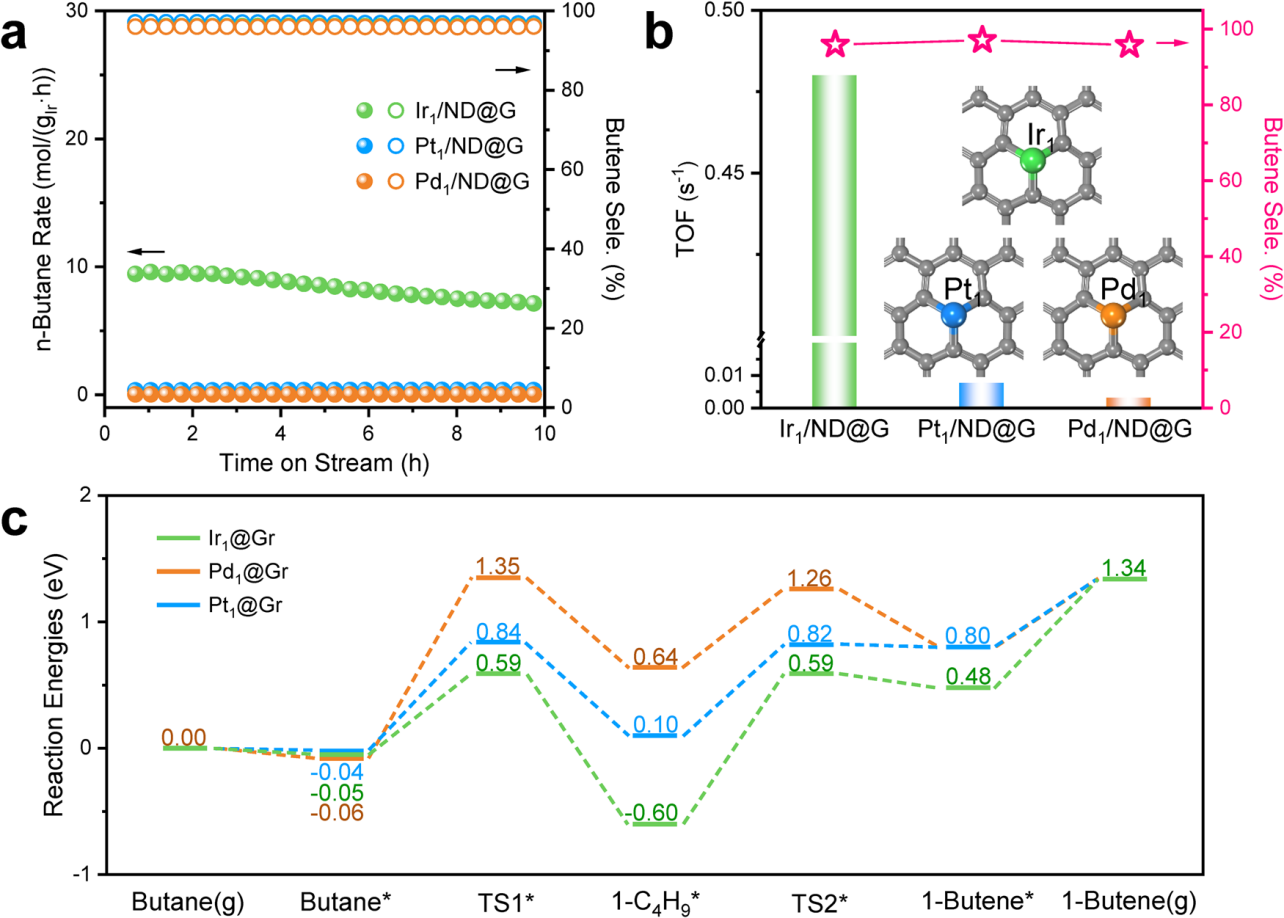
Reaction performance for BDH. **a** n-Butane reaction rate and butene selectivity of Ir_1_/ND@G, Pt_1_/ND@G, and Pd_1_/ND@G. **b** The value of TOF and butene selectivity. **c** Energy profiles of BDH on Ir_1_@Gr, Pt_1_@Gr, and Pd_1_@Gr.

As shown in Fig. 4, Supplementary Fig. 24, and Table 3, the results of catalytic performance over Ir_1_/ND@G, Pt_1_/ND@G, and Pd_1_/ND@G were summarized. The Ir_1_/ND@G containing only 0.02 wt% Ir showed a higher conversion of 4.6% than Pt_1_/ND@G (1.2%) (Supplementary Fig. 24). Moreover, the reaction rate of n-butane on Ir_1_/ND@G was 26 times higher than that of Pt_1_/ND@G (0.34 mol·g_Ir_^−1^·h^−1^) (Fig. 4a). Unexpectedly, the reaction rate of n-butane on Pd_1_/ND@G was 0.0489 mol·g_Ir_^−1^·h^−1^ (Fig. 4a), suggesting its relative inertness in C-H activation. The intrinsic activity of the above three samples was evaluated by normalizing the activity to metal atoms exposed on the surface and obtaining TOF (Fig. 4b). On Ir_1_/ND@G, the TOF reached 0.48 s^−1^, which was 62 times and 155 times higher than that of Pt_1_/ND@G and Pd_1_/ND@G respectively. To reveal the nature of the different metals´ dehydrogenation activity of n-butane, temperature-programmed surface reactions (TPSR) with the mixture of n-butane and D_2_ were also conducted on the above three samples. Here, the combination of H or D atoms to generate H_2_ or HD is assumed as a facile step in BDH. And the generation of HD indicates the first step of C−H activation. On Ir_1_/ND@G catalyst, the appearance of HD was observed at 536 K (Supplementary Fig. 25), which was more favorable than that on Pt_1_/ND@G (645 K) and Pd_1_/ND@G (670 K). The results suggest that activation of the first C-H bond on Ir_1_/ND@G is easier than that on Pt_1_/ND@G and Pd_1_/ND@G. Moreover, the onset temperature of butene formation was in the order of Ir_1_/ND@G (669 K) < Pt_1_/ND@G (745 K) < Pd_1_/ND@G (763 K), suggesting that cleavage of the second C-H bond also influence the dehydrogenation activity. Combined with the above observation, C-H activation is a rate-determining step on SACs. Both the first and second steps of C-H activation can limit dehydrogenation activity in the whole BDH process. Moreover, C-H activation is significantly dependent on the metal species. BDH activity is in the order of Ir_1_/ND@G > Pt_1_/ND@G > Pd_1_/ND@G.

**Fig. 5:**
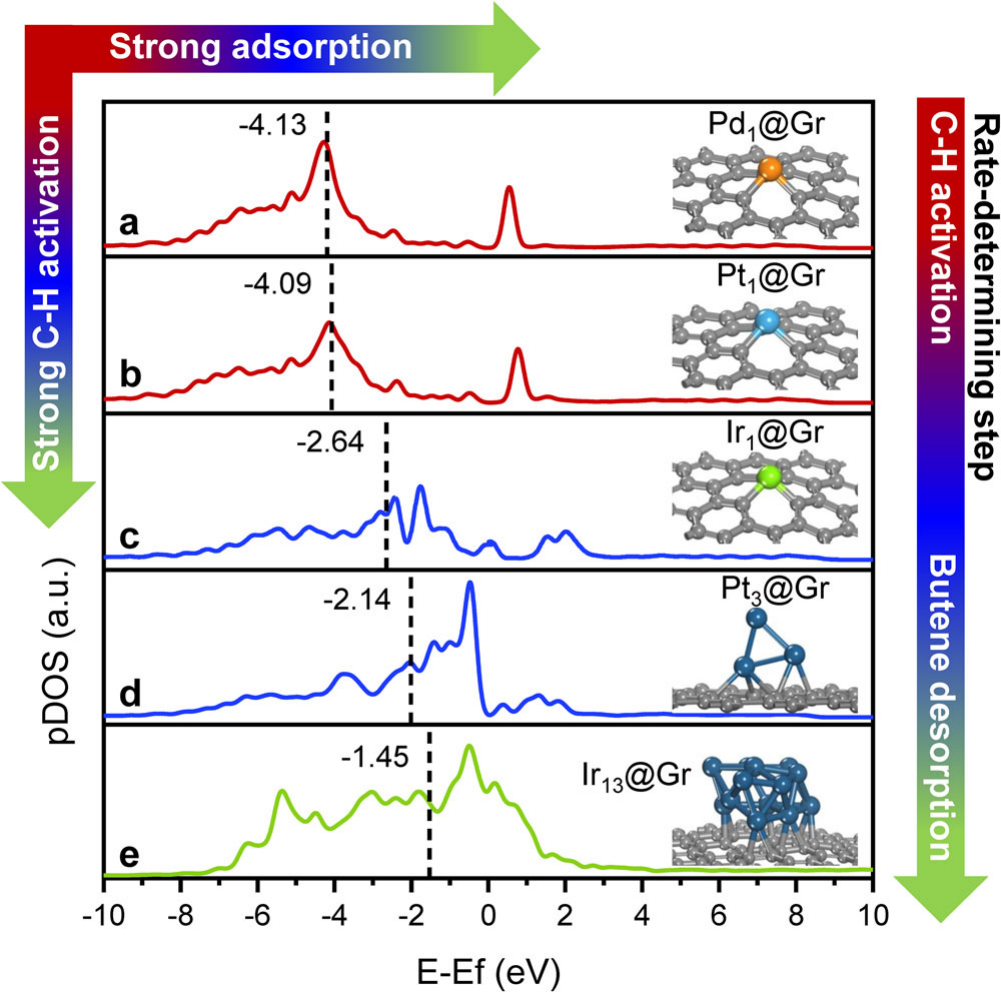
DOS curves for d orbitals of Pd, Pt, and Ir atom. The d-band center was highlighted by a dotted line. **a**–**e** DOS curve was for Pd_1_@Gr, Pt_1_@Gr, Ir_1_@Gr, Pt_3_@Gr, and Ir_13_@Gr, respectively. The *d*-band center was −4.13, −4.09, −2.64, −2.14, and −1.45 eV, respectively. The structure of these models was also presented. The arrow in the upper left represents the trend of adsorption strength and C-H activation. The arrow in the right represents the trend of shifted rate-determining step.

To gain further mechanistic insight into metal-dependent BDH activities, DFT calculations were performed on Ir_1_@Gr, Pt_1_@Gr, and Pd_1_@Gr (Supplementary Figs. 22, 26). As shown in Fig. 4c, the BDH into butene has a determining step of butane*→1-C_4_H_9_* on Pt_1_@Gr and Pd_1_@Gr, which shows a barrier of 1.41 and 0.88 eV, respectively. On Ir_1_@Gr, there is a determining step of 1-C_4_H_9_* → 1-butene* with a barrier of 1.19 eV. However, on Pt_1_@Gr, the dehydrogenation of 1-C_4_H_9_* into 1-butene* has a barrier of 0.82 eV and is endothermic by 0.80 eV, indicating that the formation of 1-butene* is unstable and easy to hydrogenate into 1-C_4_H_9_*. Compared with Pt_1_@Gr and Pd_1_@Gr, butane* dehydrogenation into 1-C_4_H_9_* has a lower barrier (0.64 vs. 0.88 and 1.41 eV) and more exothermicity (−0.55 vs. 0.14 and 0.70 eV) on Ir_1_@Gr. Thus, the formation of 1-C_4_H_9_* was favored thermodynamically and kinetically. An accumulation in the content of 1-C_4_H_9_* on Ir_1_@Gr can be predicted and will increase the possible formation of 1-butene*. In addition, the calculated *d*-band centers for Ir_1_@Gr, Pt_1_@Gr, and Pd_1_@Gr are −2.64, −4.09, and −4.13 eV, respectively (Fig. 5). This result suggests stronger adsorption will occur on Ir_1_@Gr than Pt_1_@Gr and Pd_1_@Gr, which is consistent with Fig. 4c. Based on the experimental observations and theoretical calculations, the adsorption of intermediates is depended on the metal species. The stronger adsorption of intermediates is the key to the higher activity on Ir SAs than that of Pt SAs or Pd SAs.

On Pt_1_/ND@G, the poorer activity for dehydrogenation steps was influenced by the weaker adsorption of intermediates. We propose that a higher activity on Pt would require more unsaturated Pt sites. The triangular Pt_3_ cluster (Pt_3_/ND@G) reported in our previous work was an efficient catalyst in BDH^43^. The n-butane reaction rate on Pt_3_/ND@G was nearly six times higher than that on Pt_1_/ND@G. The reason for enhanced activity would attribute to the adsorption sites with more unsaturation. The calculated *d*-band center for Pt_3_@Gr was −2.14 eV, indicating that stronger adsorption will occur on Pt_3_@Gr in contrast with Pt_1_@Gr (−4.09 eV) (Fig. 5). Moreover, the *d*-band center for Pt_3_@Gr was close to Ir_1_@Gr (−2.64 eV) and located near Fermi level, indicating the stronger adsorption of intermediates and products. The TOF of Pt_3_/ND@G (0.12 h^−1^) was four times lower than Ir_1_/ND@G (0.48 h^−1^), indicating that the rate-determining step on Pt_3_@Gr would shift from C-H activation towards butene desorption due to the stronger adsorption of products. According to the above proposal, we would further improve the catalytic activity on Ir_1_ND@G, if constructing one cluster with more unsaturated sites. Unexpectedly, the calculated *d*-band center for Ir_13_@Gr was −1.45 eV and closer to the Fermi level, compared with Ir_1_@Gr and Pt_3_@Gr (Fig. 5). Owing to the strong adsorption behavior, rate-determining step on Ir_13_@Gr located at the step of butene desorption. The difficult desorption of butene led to poor catalytic performance in BDH (Fig. 3c). Furthermore, temperature-programmed desorption of butene (C_4_H_8_-TPD) and TGA have been carried out to solidify the above conclusion from DFT calculations (Supplementary Fig. 27 and Table 4). Therefore, the highly efficient catalyst would not be determined by a more unsaturated structure but by moderate strength of adsorption, strongly dependent on the metal species. The Ir_1_ND@G with moderate adsorption behavior showed the highest catalytic performance with moderate activity and stability. Besides BDH, the nature of structure-dependence and metal-dependence have been expanded to PDH. A similar discipline has been found. For the nature of structure-dependence, Ir_1_ND@G showed higher activity than Ir_n_/ND@G and IrNPs/ND@G (Supplementary Fig. 28). For the nature of metal-dependence, Ir_1_ND@G showed high activity, and Pt_1_/ND@G and Pd_1_/ND@G was almost inactive for PDH (Supplementary Fig. 29).

## Discussion

In summary, a series of highly dispersed Ir catalysts were successfully synthesized on ND@G support for BDH, inspired by single-site pincer-ligated iridium complexes in homogeneous catalysis. To gain insights into the nature of structure-dependence and metal-dependence of catalyst, we correlated the BDH activity with different Ir structures ranging from nanoscale to sub-nanoscale and also compared the Ir SAs catalyst with Pt SAs and Pd SAs. Compared to Ir sub-nanoclusters and NPs, Ir SAs showed the highest n-butane conversion and the best butene selectivity (against both side reactions and coke formation). The suitable strength of intermediates adsorption shifted the rate-determining step from butene desorption to C-H activation. And thus, moderate dehydrogenation activity and easy desorption of 1-butene ensure the stability and outstanding catalytic performance of Ir SAs. Moreover, the adsorption of intermediates is also determined by the metal species, which is the main reason for the higher activity on Ir SAs than that of Pt SAs or Pd SAs. Our results illustrate that the isolated metal sites, not just the ensemble sites, can be adaptable toward both reactant adsorption/activation and product desorption. The good dehydrogenation capacity and moderate adsorption behavior are the key to the high catalytic activity and butene selectivity, which are strongly dependent on metal structure and content. Importantly, such nature of structure-dependence and metal-dependence pave a new path to design catalysts with high activity, selectivity, and stability for efficient dehydrogenation.

## Methods

### Materials

Nanodiamond (ND) powders were purchased from Beijing Grish Hitech Co., Ltd, China. Analytical grade Chloroiridic acid (H_2_IrCl_6_ ∙ 6H_2_O) as metal precursors were purchased from Aladin Chemical Reagent Inc. Sodium formate (HCOONa) and ammonium hydroxide aqueous solution (NH_4_OH, 25–28%) were purchased from Sinopharm Chemical Reagent Co. Ltd. China).

### Preparation of ND@G

Nanodiamond powder was annealed at 1100 °C under Ar flow (100 mL/min) for 4 h and then cooled to room temperature. The obtained ND@G powder was further purified to remove impurities by concentrated hydrochloric acid for 24 h, and then washed with deionized water. Finally, the purified ND@G was dried in a vacuum at 80 °C for 48 h.

### Preparation of Ir_1_/ND@G and IrNPs/ND@G

The catalysts were prepared by the impregnation method. Typically, a certain amount of H_2_IrCl_6_ ∙ 6H_2_O solution (10 g/L) was added into 2 mL ethanol, and the mixture was ultrasonically treated for 5 min. Then, 200 mg purified ND@G powder was added to the above solution, and the mixture was ultrasonically treated for 5 min to obtain a homogenous suspension. The mixture was stirred at room temperature for 24 h and then dried at 60 °C for another 12 h. Finally, the Ir_1_/ND@G sample was reduced in H_2_ (10 vol% H_2_ in N_2_, flow rate = 30 mL/min) at 200 °C for 1 h, and the IrNPs /ND@G sample was reduced in H_2_ (10 vol% in N_2_, flow rate = 30 mL/min) at 450 °C for 2 h. Limited by the wet chemistry synthesis method, the maximum Ir loading of SA is 0.1%, confirmed by ICP-OES. Benefiting from the low loading amount, many high-quality works reported important results in fundamental research^44–48^. Herein, we chose the Ir_1_/ND@G with 0.02 wt% Ir as the major sample.

### Preparation of Ir_1+n_/ND@G and Ir_n_/ND@G

The catalysts were prepared by the deposition-precipitation method. Typically, 200 mg purified ND@G was dispersed into 25 mL deionized water in a 100 mL round-bottom flask, and the mixture was ultrasonically treated for 30 mi. Then, the pH value of the above suspension was adjusted to about 10 by the addition of HCOONa. Afterward, a certain amount of H_2_IrCl_6_ ∙ 6H_2_O solution (10 g/L) was introduced into the above suspension, and then adjusted the pH value to 7. The mixture was stirred at 100 °C in an oil bath for 1 h. And then, the obtained mixture was cooled to room temperature, washed with deionized water, and dried in a vacuum at 60 °C for 12 h. Finally, the solid sample was reduced in H_2_ (10 vol% H_2_ in N_2_, flow rate = 30 mL/min) at 450 °C for 2 h.

### Catalytic performance

The catalytic performance for the BDH was conducted in a fixed-bed stainless steel micro-reactor with a quartz lining under atmosphere pressure at 450 °C with 20 mg catalysts. A gas mixture of 2% H_2_ and 2% n-C_4_H_10_ with He balance (flow rate = 15 mL min^−1^, GHSV = 45,000 mL g^−1^ h^−1^) was introduced. The effluent mixture gas was analyzed by online gas chromatography (Agilent 7890 with an FID and a TCD detector).

The n-butane conversion, C_4_ olefin (butene and 1, 3-butadiene) selectivity, butene selectivity, and n-butane reaction rate were calculated using the following equations:

1 $${{{{{\rm{n}}}}}}{\mbox-{{{{{{\rm{Butane}}}}}}}} \, {{{{{\rm{conversion}}}}}}=\frac{{{{{{\rm{Mole}}}}}} \, {{{{{\rm{of}}}}}} \, {{{{{\rm{the}}}}}} \, {{{{{\rm{reacted}}}}}} \, {{{{{\rm{n}}}}}}{\mbox-{{{{{{\rm{butane}}}}}}}}}{{{{{{\rm{Mole}}}}}} \, {{{{{\rm{of}}}}}} \, {{{{{\rm{inlet}}}}}} \, {{{{{\rm{n}}}}}}{\mbox-{{{{{{\rm{butane}}}}}}}}}\times 100\%$$

2 $${{{{{{\rm{C}}}}}}}_{4} \, {{{{{\rm{olefin}}}}}} \, {{{{{\rm{Selectivity}}}}}}=\frac{{{{{{\rm{Mole}}}}}} \, {{{{{\rm{of}}}}}}({{{{{\rm{butene}}}}}} \, {{{{{\rm{formed}}}}}}+1,\, 3 {\mbox-{{{{{{\rm{butadiene}}}}}}}} \, {{{{{\rm{formed}}}}}})}{{{{{{\rm{mol}}}}}} \, {{{{{\rm{of}}}}}} \, {{{{{\rm{reacted}}}}}}}\times 100\%$$

3 $${{{{{\rm{Butene}}}}}} \, {{{{{\rm{selectivity}}}}}}=\frac{{{{{{\rm{Mole}}}}}} \, {{{{{\rm{of}}}}}}\,({{{{{\rm{butene}}}}}} \, {{{{{\rm{formed}}}}}})}{{{{{{\rm{mol}}}}}} \, {{{{{\rm{of}}}}}} \, {{{{{\rm{reacted}}}}}}}\times 100\%$$

4 $${{{{{\rm{n}}}}}}{\mbox-{{{{{{\rm{Butane}}}}}}}} \, {{{{{\rm{reaction}}}}}} \, {{{{{\rm{rate}}}}}}=\frac{{{{{{\rm{Flow}}}}}} \, {{{{{\rm{rate}}}}}} \, {{{{{\rm{of}}}}}} \, {{{{{\rm{n}}}}}}{\mbox-{{{{{{\rm{butane}}}}}}}}\times {{{{{\rm{conversion}}}}}}\times 60}{{{{{{\rm{Ir}}}}}} \, {{{{{\rm{weight}}}}}}\times 22.4}$$

The n-butane conversion adapted to calculate turnover frequency (TOF) was below 15%. TOF of the catalysts was calculated using the following equation:

5 $${{{{{\rm{TOF}}}}}}=\frac{{{{{{\rm{Mole}}}}}} \, {{{{{\rm{of}}}}}} \, {{{{{\rm{n}}}}}}{\mbox-{{{{{{\rm{butane}}}}}}}} \, {{{{{\rm{conved}}}}}} \, {{{{{\rm{per}}}}}} \, {{{{{\rm{second}}}}}}}{{{{{{\rm{Mole}}}}}} \, {{{{{\rm{of}}}}}} \, {{{{{\rm{active}}}}}} \, {{{{{\rm{metal}}}}}}\times {{{{{\rm{dispersion}}}}}}}$$

The catalyst stability was described by a first-order deactivation model:

6 $${{{{{{\rm{k}}}}}}}_{{{{{{\rm{d}}}}}}}=\frac{{{{{{\rm{ln}}}}}}\left(\frac{1-{{{{{{\rm{C}}}}}}}_{{{{{{\rm{f}}}}}}}}{{{{{{{\rm{C}}}}}}}_{{{{{{\rm{f}}}}}}}}\right)-{{{{{\rm{ln}}}}}}\left(\frac{1-{{{{{{\rm{C}}}}}}}_{{{{{{\rm{i}}}}}}}}{{{{{{{\rm{C}}}}}}}_{{{{{{\rm{i}}}}}}}}\right)}{{{{{{\rm{t}}}}}}}$$

where C_i_ is the initial conversion after reaction 0.33 h; C_f_ is the final conversion after reaction 9.75 h; *t* represents the reaction time (h); and *k*_d_ is the deactivation rate constant (h^−1^) that is used to evaluate the catalyst stability (the higher *k*_d_ value is, the lower the stability).

The catalytic performance for the PDH was conducted in a fixed-bed stainless steel micro-reactor with a quartz lining under atmosphere pressure at 500 °C with 50 mg catalysts. A gas mixture of 5% C_3_H_8_ with He balance (flow rate = 15 mL min^−1^, GHSV = 18,000 mL g^−1^ h^−1^) was introduced. The effluent mixture gas was analyzed by online gas chromatography (Agilent 7890 with an FID and a TCD detector).

The propane conversion, propene selectivity, and TOF were calculated using the following equations:

7 $${{{{{\rm{Propane}}}}}} \, {{{{{\rm{conversion}}}}}}=\frac{{{{{{\rm{Mole}}}}}} \, {{{{{\rm{of}}}}}} \, {{{{{\rm{the}}}}}} \, {{{{{\rm{reacted}}}}}} \, {{{{{\rm{propane}}}}}}}{{{{{{\rm{Mole}}}}}} \, {{{{{\rm{of}}}}}} \, {{{{{\rm{inlet}}}}}} \, {{{{{\rm{propane}}}}}}}\times 100\%$$

8 $${{{{{\rm{Propene}}}}}} \, {{{{{\rm{selectivity}}}}}}=\frac{{{{{{\rm{Mole}}}}}} \, {{{{{\rm{of}}}}}}\,({{{{{\rm{propene}}}}}} \, {{{{{\rm{formed}}}}}})}{{{{{{\rm{mol}}}}}} \, {{{{{\rm{of}}}}}} \, {{{{{\rm{reacted}}}}}}}\times 100\%$$

The propane conversion adapted to calculate TOF was below 10%. TOF of the catalysts was calculated using the following equation:

9 $${{{{{\rm{TOF}}}}}}=\frac{{{{{{\rm{Mole}}}}}} \, {{{{{\rm{of}}}}}} \, {{{{{\rm{propane}}}}}} \, {{{{{\rm{conved}}}}}} \, {{{{{\rm{per}}}}}} \, {{{{{\rm{second}}}}}}}{{{{{{\rm{Mole}}}}}} \, {{{{{\rm{of}}}}}} \, {{{{{\rm{active}}}}}} \, {{{{{\rm{metal}}}}}}\times {{{{{\rm{dispersion}}}}}}}$$

### Regeneration tests

After the initial run, the catalyst was calcinated in 20% O_2_ (30 mL/min) at 300 °C for 1.5 h and reduced in pure H_2_ at 500 °C for 4 h to remove O sites. Finally, a gas mixture of 2% H_2_ and 2% n-C_4_H_10_ with He balance (flow rate = 15 mL min^−1^, GHSV = 30,000 mL g^−1^ h^−1^) was introduced, regarded as one regeneration cycle.

### Catalyst characterization

HRTEM images were taken by an FEI Tecnai G2 F20 working at 200 kV. HAADF-STEM images were recorded by a JEOL JEM ARM 200CF aberration-corrected cold field-emission scanning transmission electron microscope at 200 kV. The sample powder has been dispersed into ethanol and ultrasonically treated for 5 min to obtain a homogenous suspension. And then, we extracted the supernatant of 10 μL and dropped it onto holey carbon-coated copper grids in the atmosphere. XPS were carried out at ESCALAB 250 instrument with Al Kα X-rays (1489.6 eV, 150 W, 50.0 eV pass energy) and the C 1*s* peak at 284.6 eV as internal standard. Before XPS characterization, the samples were reduced by 10% H_2_ and collected in the atmosphere. XRD patterns were obtained by using a D/MAX-2500 PC X-ray diffractometer with monochromatized CuKα radiation (λ = 1.54 Å). Before XRD characterization, the samples were reduced by 10% H_2_ and collected in the atmosphere. In situ CO-DRIFTS over different Ir catalysts were recorded on a Thermo Scientific Nicolet IS10 Fourier transform infrared spectrometer equipped with a high-temperature and high-pressure chamber and an MCT detector. Before measurement, the unreduced samples were diluted with KBr were placed in the sample holder, and reduced in H_2_ (5 mL/min) for 1 h at the desired reduction temperature. And then, the sample holder was cooled to 293 K under He atmosphere. CO adsorption was carried out at room temperature in a flow of 5%CO/He (5 mL/min). Until the surface is completely covered by CO, the gas was switched to He (5 mL/min), and the spectrum was continuously recorded at 293 K. For in situ CO-DRIFTS of Ir_1_/ND@G under reaction condition, the samples with KBr were placed in the sealed chamber and reduced in 10% H_2_ (5 mL/min) for 1 h at 200 °C. After the chamber was heated to 450 °C in He, the gas was switched to reaction gas (2% C_4_H_10_, 2% H_2_, He balance) and treated for 1 h at the reaction condition. And then, the sealed chamber was cooled to 20 °C in reaction gas. CO adsorption was carried out at 20 °C in a flow of 5% CO/He (5 mL/min). Until the surface is completely covered by CO, the gas was switched to He (5 mL/min) and the spectrum was continuously recorded at 20 °C. Furthermore, in order to evaluate the structure of the Ir_1_/ND@G catalyst even in the H_2_ atmosphere under the reaction temperature, the reduction temperature was raised to 450 °C (reaction temperature). For in situ CO-DRIFTS of Ir_1_/ND@G under reduction treatment, the samples with KBr were placed in the sealed chamber and reduced in 10% H_2_ (5 mL/min) for 1 h at 200 °C. The chamber was heated to 450 °C in He after reduction, the gas was switched to 10% H_2_ and treated for 1 h at 450 °C. And then, the sealed chamber was cooled to 20 °C. CO adsorption was carried out at 20 °C in a flow of 5% CO/He (5 mL/min). Until the surface is completely covered by CO, the gas was switched to He (5 mL/min) and the spectrum was continuously recorded at 20 °C. The Brunauer−Emmett−Teller (BET) surface area, BJH pore volume, and average pore diameter of the as-prepared samples were measured by N_2_ physisorption on the Micrometrics ASAP-2020 instrument. Before BET characterization, the samples were reduced by 10% H_2_ and collected in the atmosphere. Elemental analysis of Iridium in the solid catalysts was detected by ICP-OES analysis. Before ICP-OES characterization, the samples were reduced by 10% H_2_ and collected in the atmosphere. XAFS measurement at Ir *L*_3_-edge (11,215 eV) was measured at the beamline BL14W1 station in Shanghai Synchrotron Radiation Facility (SSRF). The focused beam was tuned by the Si (111) double-crystal monochromators. Ir foil and IrO_2_ were used as standards. The samples were measured in fluorescence mode, using a Lytle detector to collect the data. For the quasi-in situ XAS spectra of reduced catalysts, the samples were reduced in a fixed-bed reactor with 10 vol% H_2_/N_2_ at corresponding temperatures. After it was cooled to atmospheric temperature, the reactor was sealed with two globe valves and transferred to the glove box without exposure to air. And then, the prepared catalysts were sealed in a measurement plate by Kapton films under Ar protection. The whole process was performed in the glove box. The reduced sample could not be oxidized by this sample preparation method for XAS data collection. For Ir_1_/ND@G, a 32-channel solid detector was to achieve high data quality. All XAFS spectra were processed and analyzed by the Ifeffit package. The amount of carbon deposition was measured through the combustion of spent Ir/ND@G catalysts on the model STA 409 PC/PG thermogravimetric analyzer (NETZSCH). After BDH, the samples were collected in the air. UV-Raman spectroscopy was performed on powder samples by using the HORIBA LabRam HR Raman spectrometer, and the excitation wavelength was 325 nm with a power of 0.2 mW (exposure 20 s, accumulated four times). Before UV-Raman characterization, the samples before or after BDH were prepared in the air. In situ TEM is a powerful tool to verify the real structure of active species under a reaction atmosphere. However, due to the dramatically increased electron scattering from gas-cell membranes (usually two 50nm-thick SiN films both above and beneath the 5–6-nm-thick nanodiamond sample) and from the H_2_ atmosphere, as well as the thermal turbulence in 450 °C high temperature, the originally weak signal of single Ir atoms cannot be detected, considering TEM capacities. For in situ XAFS, the low loading amount (0.02 wt%) and the harsh reaction condition (450 °C) also lead to a noisy and weak signal of single Ir atoms. It is difficult to detect and analyze the precise structure information. Therefore, current eTEM and in situ XAFS techniques cannot provide the solid but desired evidence on the structure of Ir_1_/ND@G under reaction conditions. To verify the catalytic structure, the HAADF-STEM images of the Ir_1_/ND@G after 0.5 h BDH at 450 °C have been provided (Supplementary Fig. 18).

### Temperature-programmed surface reaction (TPSR)

The TPSR experiments for the BDH were conducted in a fixed-bed reactor with a quartz lining under the atmosphere. About 10 mg SACs were set in a 4 mm diameter quartz tube and were reduced in D_2_ (10% D_2_/Ar, flow rate = 20 mL/min) at 473 K for 2 h. About 10 mg Ir_n_ and IrNPs catalysts were reduced in D_2_ (10% D_2_/Ar, flow rate = 20 ml/min) at 723 K for 2 h. After the catalyst was cooled to room temperature, the gas was switched to 10% n-C_4_H_10_/Ar (flow rate = 20 mL/min) and 10% D_2_/Ar (flow rate = 2 mL/min), and the purging was continued for two hours to stabilize the signal. Then TPSR was operated at the programmed rising temperature for 5 K/min. A mass detector (OMNI StarTM GSD 350) was used to analyze the signal of m/z = 2 (H_2_), 3 (HD), 4 (D_2_), 56 (C_4_H_8_), and 58 (C_4_H_10_).

### Temperature-programmed desorption (TPD)

Temperature-programmed desorption of butene (C_4_H_8_-TPD) were performed in a quartz-bed flow reactor with an online mass spectrometer (MS, Pfeiffer OMNIstarTM). Typically, 50 mg samples were reduced in H_2_ (10 % H_2_/He, flow rate = 20 mL/min) at 200 °C for 2 h and treated in He (20 mL/min) to remove surface H species until the catalyst was cooled to room temperature. Then, the gas was switched to a flowing 2% C_4_H_8_/He (20 mL/min) and treated at room temperature for 2 h. After that, the system was swept in a flowing He streams (20 min mL/min) until a stable baseline was obtained. The temperature of the catalyst was then increased from 20 to 420 °C at the programmed rising temperature for 5 °C/min.

### Computational details

All DFT calculations with Perdew–Burke–Ernzerhof (PBE) funcitional^49^ were performed by Vienna ab initio simulation package (VASP)^50,51^. The projector augmented wave (PAW)^52,53^ potential was used to describe the interaction between ion and electron. A cutoff energy is set by 500 eV, the convergence tolerance is 10^−5^ eV and 0.02 eV/Å for electronic and ionic optimizations, respectively. By removing a carbon atom, there forms a carbon defect on a graphene layer (5 × 5). Pd_1_/ND@G, Ir_1_/ND@G, and Pt_1_/ND@G models are constructed by placing metal atoms at a carbon defect of a graphene layer. On the basis of EXAFS data, a two-layer Ir_13_ cluster is doped in the carbon defect to construct an Ir_13_/ND@G model. A *p*(3 × 3) Ir(111) surface with four layers was modeled and the top layers were fully relaxed as well as the bottom layers were fixed. The vacuum layers are set by 20 Å. The Brillouin zone was sampled with a grid of 3 × 3 × 1 Monkhorst Pack grid^54^. The Gaussian smearing method was used with a smearing width of 0.05 eV. Constrained scan^55^ combined with a DIMER method^56^ were used to search the transition states, which were confirmed with only one imaginary frequency by frequency calculations. Gibbs free energies at 723 K and 1 atm were corrected with the VASPKIT mode^57^.

## Supplementary information

Supplementary Information

Peer Review File

## Data Availability

The data supporting this article and other findings are available from the corresponding authors upon request. Source data are provided with this paper.

## Source data

Source Data

## References

[1] Choi J, MacArthur AHR, Brookhart M, Goldman AS (2011). Dehydrogenation and related reactions catalyzed by iridium pincer complexes. Chem. Rev..

[2] Goldman AS (2006). Catalytic alkane metathesis by tandem alkane dehydrogenation olefin metathesis. Science.

[3] Yao W, Zhang Y, Jia X, Huang Z (2014). Selective catalytic transfer dehydrogenation of alkanes and heterocycles by an iridium pincer complex. Angew. Chem. Int. Ed..

[4] Das K, Kumar A (2019). Chapter one - Alkane dehydrogenation reactions catalyzed by pincer-metal complexes. Adv. Organomet. Chem..

[5] Crabtree RH, Mihelcic JM, Quirk JM (1979). Iridium complexes in alkane dehydrogenation. J. Am. Chem. Soc..

[6] Liu FC, Pak EB, Singh B, Jensen CM, Goldman AS (1999). Dehydrogenation of n-alkanes catalyzed by iridium “pincer” complexes: regioselective formation of alpha-olefins. J. Am. Chem. Soc..

[7] Haibach MC, Kundu S, Brookhart M, Goldman AS (2012). Alkane metathesis by tandem alkane-dehydrogenation-olefin-metathesis catalysis and related chemistry. Acc. Chem. Res..

[8] Zhang X (2020). N-bridged pincer iridium complexes for highly efficient alkane dehydrogenation and the relevant linker effects. ACS Catal..

[9] Sheludko B, Cunningham MT, Goldman AS, Celik FE (2018). Continuous-flow alkane dehydrogenation by supported pincer-ligated iridium catalysts at elevated temperatures. ACS Catal..

[10] Huang Z (2009). Highly active and recyclable heterogeneous iridium pincer catalysts for transfer dehydrogenation of alkanes. Adv. Synth. Catal..

[11] Huang Z (2010). Efficient heterogeneous dual catalyst systems for alkane metathesis. Adv. Synth. Catal..

[12] Chen X, Peng M, Xiao D, Liu H, Ma D (2022). Fully exposed metal clusters: fabrication and application in alkane dehydrogenation. ACS Catal..

[13] Hook A, Celik FE (2017). Predicting selectivity for ethane dehydrogenation and coke formation pathways over model Pt–M surface alloys with ab initio and scaling methods. J. Phys. Chem. C..

[14] Guidotti M (2006). Catalytic dehydrogenation of propane over cluster-derived Ir–Sn/SiO2 catalysts. Catal. Lett..

[15] Somerville DM, Shapley JR (1998). Zeolite-NaY-supported Ir/Sn catalysts derived from single- and dual-source organometallic precursors. Preparation and characterization of highly selective dehydrogenation catalysts. Catal. Lett..

[16] Zhou Y (2022). Peripheral-nitrogen effects on the Ru1 centre for highly efficient propane dehydrogenation. Nat. Catal..

[17] Sun G (2018). Breaking the scaling relationship via thermally stable Pt/Cu single atom alloys for catalytic dehydrogenation. Nat. Commun..

[18] Hannagan RT (2021). First-principles design of a single-atom–alloy propane dehydrogenation catalyst. Science.

[19] Yang Z (2020). Coking-resistant iron catalyst in ethane dehydrogenation achieved through siliceous zeolite modulation. J. Am. Chem. Soc..

[20] Wang W (2022). Single Co sites in ordered SiO2 channels for boosting nonoxidative propane dehydrogenation. ACS Catal..

[21] Huang Z (2023). Illustrating new understanding of adsorbed water on silica for inducing tetrahedral cobalt(II) for propane dehydrogenation. Nat. Commun..

[22] Ma R (2022). Insights into the nature of selective nickel sites on Ni/Al2O3 catalysts for propane dehydrogenation. ACS Catal..

[23] Deng Y (2022). Few-atom Pt ensembles enable efficient catalytic cyclohexane dehydrogenation for hydrogen production. J. Am. Chem. Soc..

[24] Liu Z (2021). Tuning the selectivity of catalytic nitriles hydrogenation by structure regulation in atomically dispersed Pd catalysts. Nat. Commun..

[25] Peng M (2022). Antisintering Pd1 catalyst for propane direct dehydrogenation with in situ active sites regeneration ability. ACS Catal..

[26] Yang J (2021). Highly active and stable Ir nanoclusters derived from Ir1/MgAl2O4 single-atom catalysts. J. Chem. Phys..

[27] Lu Y (2018). Identification of the active complex for CO oxidation over single-atom Ir-on-MgAl2O4 catalysts. Nat. Catal..

[28] Jin R (2019). Low temperature oxidation of ethane to oxygenates by oxygen over iridium-cluster catalysts. J. Am. Chem. Soc..

[29] Hadjiivanov KI, Vayssilov GN (2002). Characterization of oxide surfaces and zeolites by carbon monoxide as an IR probe molecule. Adv. Catal..

[30] Dong C (2020). Supported metal clusters: fabrication and application in heterogeneous catalysis. ACS Catal..

[31] Peng M (2021). Fully exposed cluster catalyst (FECC): toward rich surface sites and full atom utilization efficiency. ACS Cent. Sci..

[32] Atanasoska L, Atanasoski R, Trasatti S (1990). XPS and AES study of mixed layers of RuO_2_ and IrO_2_. Vacuum.

[33] Duckers K, Bonzel HP (1989). Core and valence level spectroscopy with y-m-zeta radiation - co and k on (110) surfaces of ir, pt and au. Surf. Sci..

[34] Wang Q (2020). Coordination engineering of iridium nanocluster bifunctional electrocatalyst for highly efficient and pH-universal overall water splitting. Nat. Commun..

[35] Xiao M (2019). A single-atom iridium heterogeneous catalyst in oxygen reduction reaction. Angew. Chem. Int. Ed..

[36] Sattler JJ, Beale AM, Weckhuysen BM (2013). Operando Raman spectroscopy study on the deactivation of Pt/Al2O3 and Pt-Sn/Al2O3 propane dehydrogenation catalysts. Phys. Chem. Chem. Phys..

[37] Fan X (2020). Mn-doping induced changes in Pt dispersion and PtxMny alloying extent on Pt/Mn-DMSN catalyst with enhanced propane dehydrogenation stability. J. Catal..

[38] Zhang W (2020). Size dependence of Pt catalysts for propane dehydrogenation: from atomically dispersed to nanoparticles. ACS Catal..

[39] Wang, L. et al. Cooperative sites in fully exposed Pd clusters for low-temperature direct dehydrogenation reaction. *ACS Catal*. **11**, 11469-11477 (2021).

[40] Liu L (2019). Regioselective generation and reactivity control of subnanometric platinum clusters in zeolites for high-temperature catalysis. Nat. Mater..

[41] Liu L (2020). Structural modulation and direct measurement of subnanometric bimetallic PtSn clusters confined in zeolites. Nat. Catal..

[42] Sun X, Han P, Li B, Zhao Z (2018). Tunable catalytic performance of single Pt atom on doped graphene in direct dehydrogenation of propane by rational doping: a density functional theory study. J. Phys. Chem. C..

[43] Chen X (2021). Regulating coordination number in atomically dispersed Pt species on defect-rich graphene for n-butane dehydrogenation reaction. Nat. Commun..

[44] Chen Y (2020). Engineering the atomic interface with single platinum atoms for enhanced photocatalytic hydrogen production. Angew. Chem. Int. Ed..

[45] Ji Y (2022). Negatively charged single-atom Pt catalyst shows superior SO2 tolerance in NOx reduction by CO. ACS Catal..

[46] Liu L (2019). Determination of the evolution of heterogeneous single metal atoms and nanoclusters under reaction conditions: which are the working catalytic sites?. ACS Catal..

[47] Sun X (2022). In situ investigations on structural evolutions during the facile synthesis of cubic alpha-MoC(1-x) catalysts. J. Am. Chem. Soc..

[48] Zhang X (2021). A stable low-temperature H2-production catalyst by crowding Pt on alpha-MoC. Nature.

[49] Perdew JP (1992). Atoms, molecules, solids, and surfaces - applications of the generalized gradient approximation for exchange and correlation. Phys. Rev. B.

[50] Kresse G, Furthmuller J (1996). Efficiency of ab-initio total energy calculations for metals and semiconductors using a plane-wave basis set. Comput. Mater. Sci..

[51] Kresse G, Furthmuller J (1996). Efficient iterative schemes for ab initio total-energy calculations using a plane-wave basis set. Phys. Rev. B.

[52] Blochl PE (1994). Projector augmented-wave method. Phys. Rev. B.

[53] Kresse G, Joubert D (1999). From ultrasoft pseudopotentials to the projector augmented-wave method. Phys. Rev. B.

[54] Monkhorst HJ, Pack JD (1976). Special points for Brillouin-zone integrations. Phys. Rev. B.

[55] Plessow PN (2018). Efficient transition state optimization of periodic structures through automated relaxed potential energy surface scans. J. Chem. Theory Comput..

[56] Henkelman G, Jonsson H (1999). A dimer method for finding saddle points on high dimensional potential surfaces using only first derivatives. J. Chem. Phys..

[57] Wang V, Xu N, Liu JC, Tang G, Geng WT (2021). VASPKIT: a user-friendly interface facilitating high-throughput computing and analysis using VASP code. Comput. Phys. Commun..

