# Supplementary material for: Structure-dependence and metal-dependence on atomically dispersed Ir catalysts for efficient n-butane dehydrogenation
Source: Nat Commun. 2023 May 5;14:2588. doi: 10.1038/s41467-023-38361-4 (PMC10162968; doi:10.1038/s41467-023-38361-4)
Supplement: Supplementary file 1 — Supplementary Information [file 41467_2023_38361_MOESM1_ESM.pdf]

Supplementary Information

Structure-dependence and Metal-dependence on Atomically Dispersed Ir Catalysts for Efficient n-Butane Dehydrogenation

Xiaowen Chen,[1, 2 #] Xuetao Qin,[3 #] Yueyue Jiao,[4, 5, 6 #] Mi Peng,[3] Jiangyong Diao,[1] Pengju Ren,[4, 5] Chengyu Li, [3] Dequan Xiao,[7] Xiaodong Wen,[4, 5] Zheng Jiang,[8] Ning Wang,[9] Xiangbin Cai,[9 *] Hongyang Liu,[1, 2 *] and Ding Ma[3 *]

[1] Shenyang National Laboratory for Materials Science, Institute of Metal Research, Chinese Academy of Sciences, Shenyang 110016, P. R. China.

[2] School of Materials Science and Engineering, University of Science and Technology of China, Shenyang 110016, P. R. China.

[3] Beijing National Laboratory for Molecular Sciences, College of Chemistry and Molecular Engineering, Peking University, Beijing 100871, P. R. China.

[4] State Key Laboratory of Coal Conversion, Institute Coal Chemistry, Chinese Academy of Sciences, Taiyuan 030001, P. R. China.

[5] National Energy Center for Coal to Clean Fuel, Synfuels China Co., Ltd, Beijing 100871, P. R. China

[6] The University of Chinese Academy of Sciences, Beijing 100049, P.R. China.

[7] Center for Integrative Materials Discovery, Department of Chemistry and Chemical and Biomedical Engineering, University of New Haven, West Haven, Connecticut 06516, United States.

[8] Shanghai Institute of Applied Physics, Chinese Academy of Sciences, Shanghai 201204, P. R. China.

[9] Department of Physics and Center for Quantum Materials, Hong Kong University of Science and Technology, Kowloon, Hong Kong SAR, P. R. China.

[#] These authors contributed equally to this work.

Corresponding Author

*Email: xcaiak@connect.ust.hk; liuhy@imr.ac.cn; dma@pku.edu.cn

Supplementary Information includes:

Supplementary Figures:

Supplementary Figure 1. HRTEM images of ND@G. (a) The surface structure at low magnification of ND@G. (b-d) The surface structure at high magnification of ND@G.

Supplementary Figure 2. Microscopic characterizations of Ir_{1+n}/ND@G. (a)-(d) HAADF-STEM images of Ir_{1+n}/ND@G. The Ir clusters were highlighted by yellow circles. (e) Particle size distribution of Ir_{1+n}/ND@G.

Supplementary Figure 3. HAADF-STEM images of Ir_n/ND@G. (a) The image of Ir_n/ND@G at low magnification. (b) and (c) The image of Ir_n/ND@G at high magnification.

Supplementary Figure 4. HAADF-STEM images of IrNPs/ND@G. (a) The image of IrNPs/ND@G at low magnification. The inset is the particle size distribution of IrNPs/ND@G. (b-d) The image of IrNPs/ND@G at high magnification.

Supplementary Figure 5. *In-situ* CO-DRIFTS of IrNPs/ND@G.

Supplementary Figure 6. Ir L_3 XANES spectra of Ir_1/ND@G, Ir foil and IrO_2.

Supplementary Figure 7. Pt 4*f* XPS spectra of Ir_{1+n}/ND@G, Ir_n/ND@G and IrNPs/ND@G.

Supplementary Figure 8. EXAFS fitting results of Ir L_3-edge in R space. (a) Ir_{1+n}/ND@G, (b) Ir_n/ND@G and (c) IrNPs/ND@G.

Supplementary Figure 9. EXAFS fitting results of Ir L_3-edge in k^3 space. (a) Ir_1/ND@G, (b) Ir_{1+n}/ND@G, (c) Ir_n/ND@G and (d) IrNPs/ND@G.

Supplementary Figure 10. The catalytic performance of as-prepared catalysts. n-Butane conversion and butene selectivity by time-on-stream during BDH at 450 ℃, GHSV = 45000 $mL \cdot g_{cat}^{-1} \cdot h^{-1}$, n-$C_4H_{10}$: H_2 = 1:1 with He balance.

Supplementary Figure 11. Microscopic characterizations of Ir catalysts after 10h BDH. HAADF-STEM images of (a and b) Ir_{1+n}/ND@G and (c and d) Ir_n/ND@G.

Supplementary Figure 12. The catalytic performance of 50mg as-prepared catalysts. n-Butane conversion and butene selectivity by time-on-stream during BDH at 450 ℃, GHSV = 18000 $mL \cdot g_{cat}^{-1} \cdot h^{-1}$, n-$C_4H_{10}$: H_2 = 1:1 with He balance.

Supplementary Figure 13. Stability test over Ir_1/ND@G at 450 ℃ for 20 hours.

Supplementary Figure 14. Stability test over Ir_1/ND@G at 450 ℃ for 50h. Reaction condition: GHSV = 30000 $mL \cdot g_{cat}^{-1} \cdot h^{-1}$, n-$C_4H_{10}$:$H_2$ = 1:1 with He balance.

Supplementary Figure 15. Raman spectra for the fresh and spent catalysts after 10-hours BDH. Raman spectra of (a) fresh Ir_1/ND@G, (b) spent Ir_1/ND@G, (c) fresh Ir_n/ND@G, (d) spent Ir_n/ND@G, (e) fresh IrNPs/ND@G and (f) spent IrNPs/ND@G.

Supplementary Figure 16. HAADF-STEM images of Ir_1/ND@G after 10h BDH. (a) The image of Ir_1/ND@G after 10h BDH at low magnification. (b) and (c) The image of Ir_1/ND@G after 10h BDH at high magnification. The red circles highlighted the small Ir clusters. The yellow circles highlighted single Ir atoms.

Supplementary Figure 17. Supplementary Figure 17. HAADF-STEM images of Ir_1/ND@G after 0.5h BDH. (a) The image of Ir_1/ND@G after 0.5h BDH at low magnification. (b-d) The image of Ir_1/ND@G after 0.5h BDH at high magnification. The red circles highlighted single Ir atoms.

Supplementary Figure 18. Supplementary Figure 18. HAADF-STEM images of Ir_1/ND@G after 20h BDH. (a) The image of Ir_1/ND@G after 20h BDH at low magnification.

(b) and (c) The image of Ir_1/ND@G after 20h BDH at high magnification. The red circles highlighted single Ir atoms.

Supplementary Figure 19. The conversion of butane and selectivity of butene over Ir_1/ND@G catalyst after several regeneration cycles. Reaction condition: atmospheric pressure, GHSV = 30000 mL · g_{cat}^{-1} · h^{-1}, C_4H_{10}:H_2 = 1:1, He balance, 450 °C.

Supplementary Figure 20. The *in-situ* CO-DRIFTS of Ir_1/ND@G under reaction condition for 1h. Reaction conditions: 450 °C, 2%C_4H_{10}, 2%H_2, He balance.

Supplementary Figure 21. The *in-situ* CO-DRIFTS of Ir_1/ND@G under reduction condition for 1h. Reduction conditions: 450 °C, 10%H_2/He.

Supplementary Figure 22. The optimized configurations of surface intermediates on Ir_1@Gr, Ir_{13}@Gr, Ir(111), Pd_1@Gr and Pt_1@Gr.

Supplementary Figure 23. Gibbs free energies profiles of BDH on Ir(111), Ir_{13}@Gr and Ir_1@Gr.

Supplementary Figure 24. n-Butane conversion and butene selectivity of Ir_1/ND@G, Pt_1/ND@G and Pd_1/ND@G.

Supplementary Figure 25. TPSR profiles of the mixture of n-butane and D_2 on the different catalysts. (a) Ir_1/ND@G, (b) Pt_1/ND@G and (c) Pd_1/ND@G.

Supplementary Figure 26. Gibbs free energies profiles of BDH on Pd_1@Gr, Pt_1@Gr and Ir_1@Gr.

Supplementary Figure 27. C_4H_8-TPD profiles of the different catalysts. (a) ND@G, (b) Ir_1/ND@G, (c) Ir_n/ND@G and (d) Pt_3/ND@G. The peak at 80 ℃ is corresponding to physical absorption on ND@G.

Supplementary Figure 28. TOF and C_3H_6 selectivity over Ir_1/ND@G, Ir_{1+n}/ND@G, Ir_n/ND@G and IrNPs/ND@G.

Supplementary Figure 29. C_3H_8 conversion over Ir_1/ND@G, Pt_1/ND@G, Pd_1/ND@G and ND@G.

Supplementary Tables:

Supplementary Table 1. Physical structure properties of the catalysts.

Supplementary Table 2. Ir L_3-edge EXAFS fitting results for as-prepared catalysts.

Supplementary Table 3. Catalytic performance of the catalysts for BDH.

Supplementary Table 4. Amount of carbon deposition on the catalysts for 10-hour BDH.

Supplementary Table 5. Catalytic performance of the catalysts for BDH.

Supplementary Table 6. Summary of the catalytic performance of various supported metal catalysts for BDH.

Supplementary References

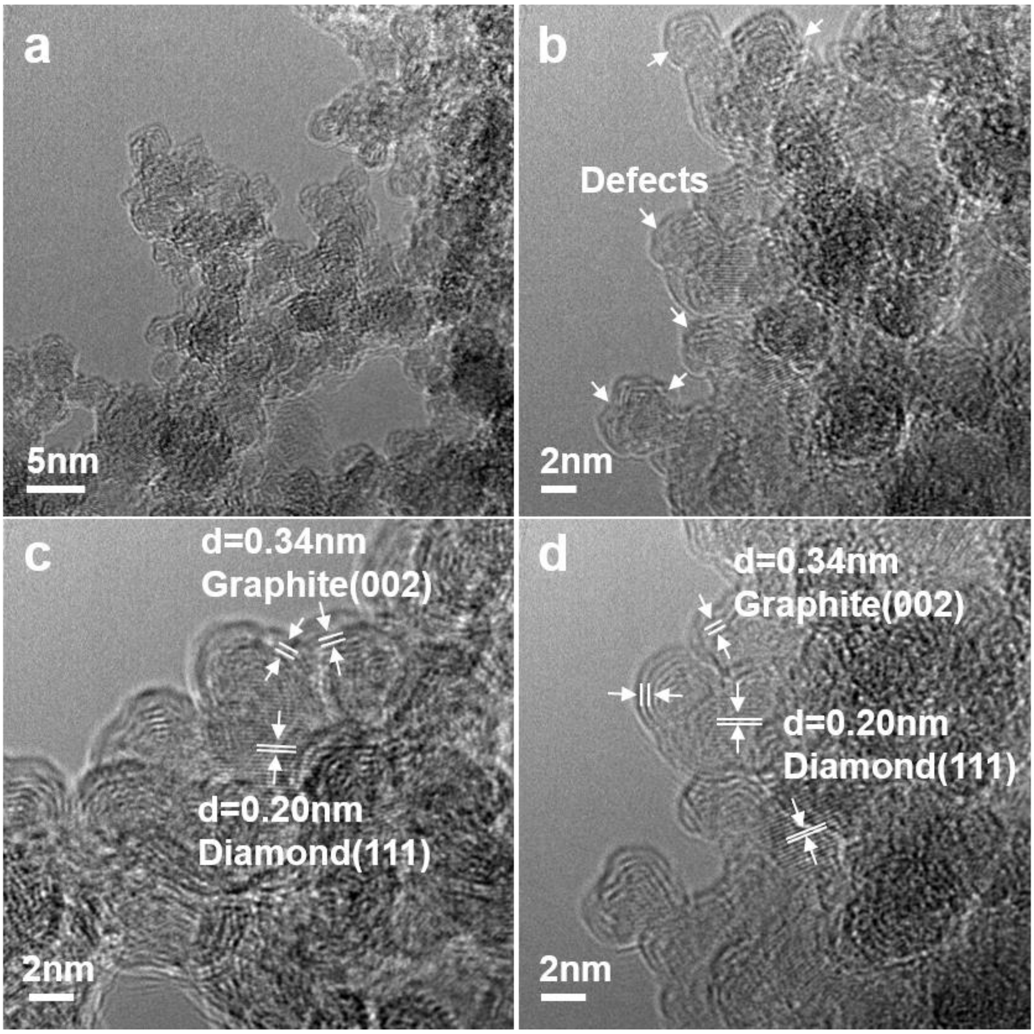

Supplementary Figure 1. HRTEM images of ND@G. (a) The surface structure at low magnification of ND@G. (b-d) The surface structure at high magnification of ND@G.

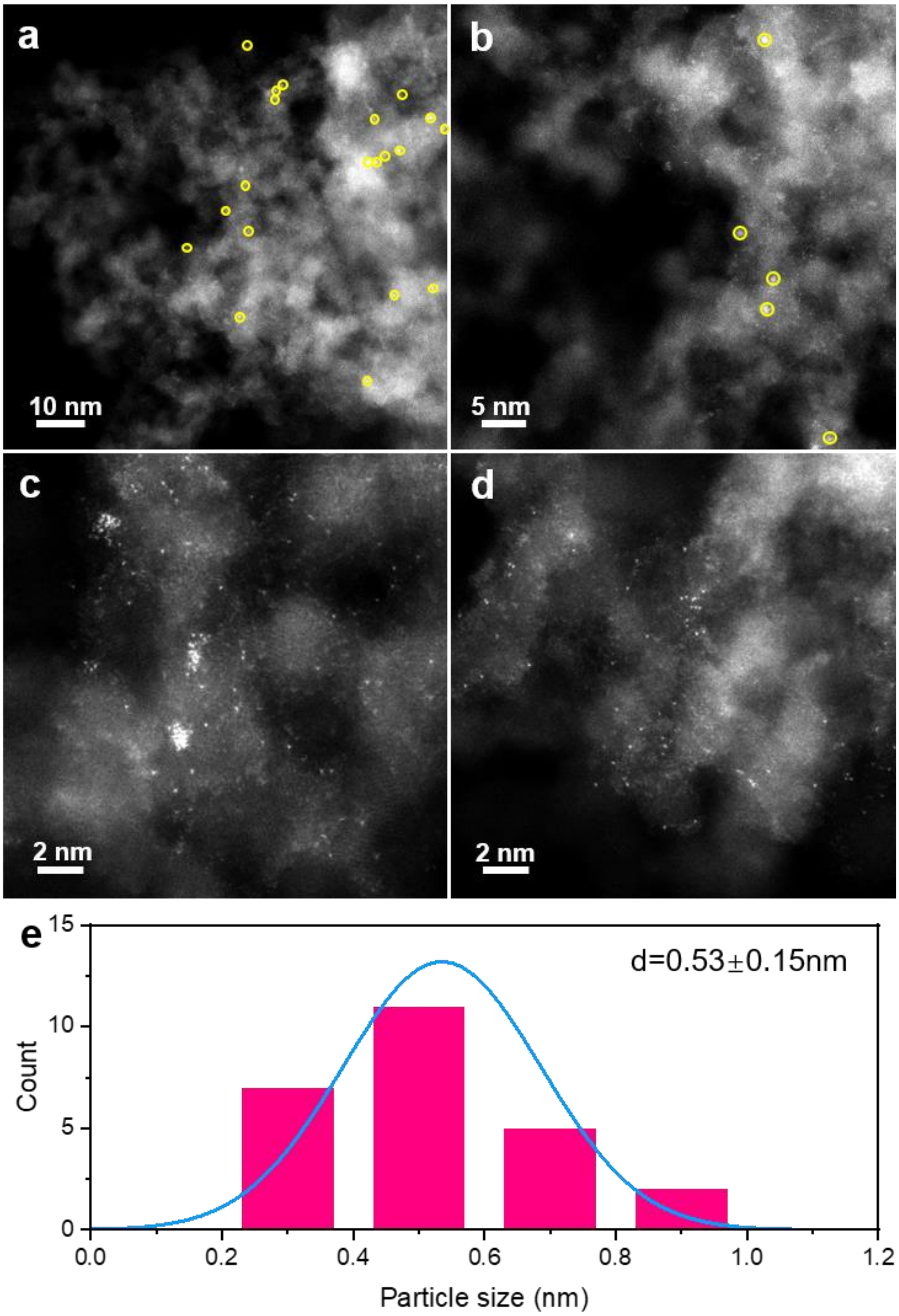

Supplementary Figure 2. Microscopic characterizations of Ir_{1+n}/ND@G. (a)-(d) HAADF-STEM images of Ir_{1+n}/ND@G. The Ir clusters were highlighted by yellow circles. (e) Particle size distribution of Ir_{1+n}/ND@G.

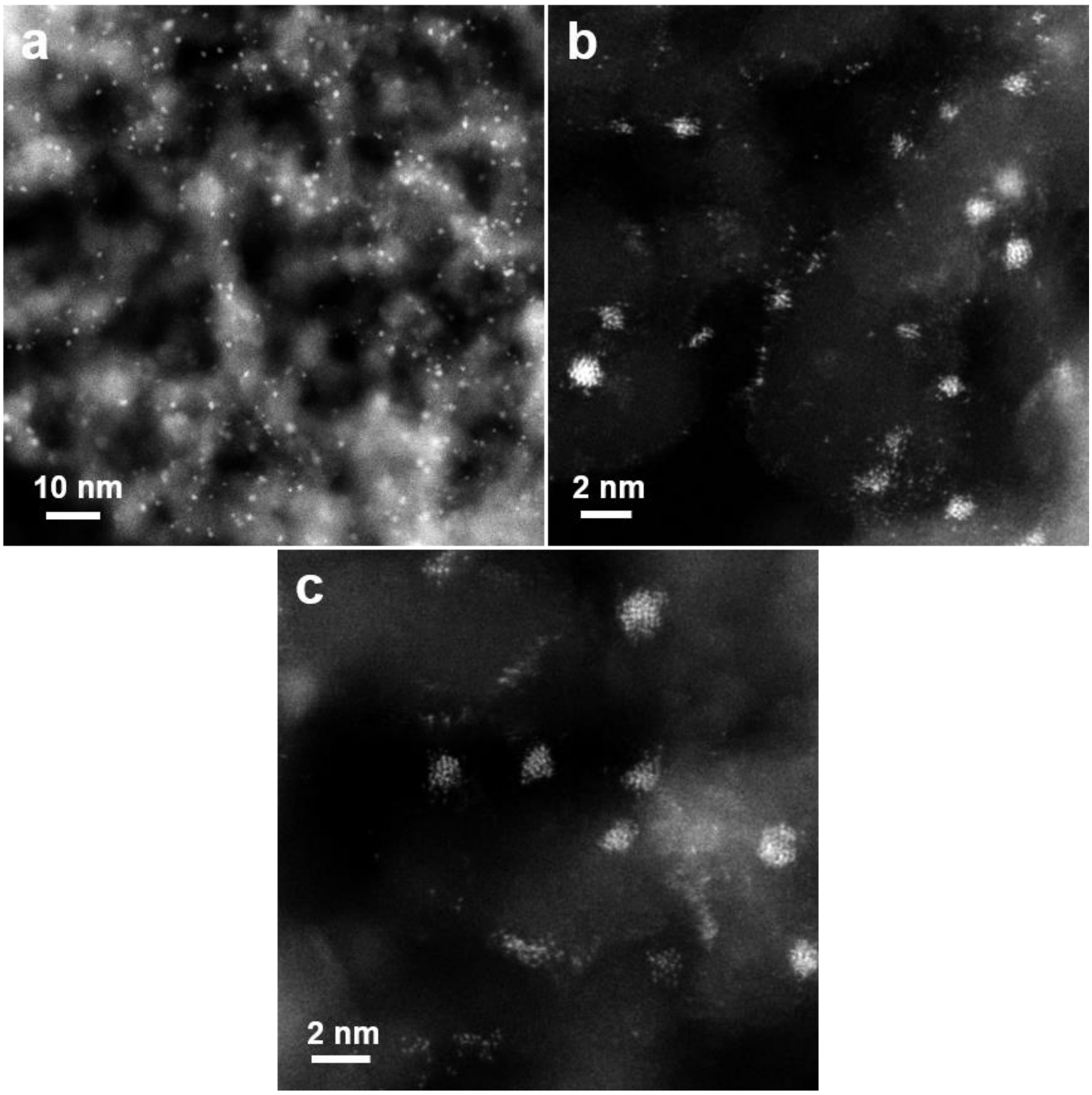

Supplementary Figure 3. HAADF-STEM images of Ir_n/ND@G. (a) The image of Ir_n/ND@G at low magnification. (b) and (c) The image of Ir_n/ND@G at high magnification.

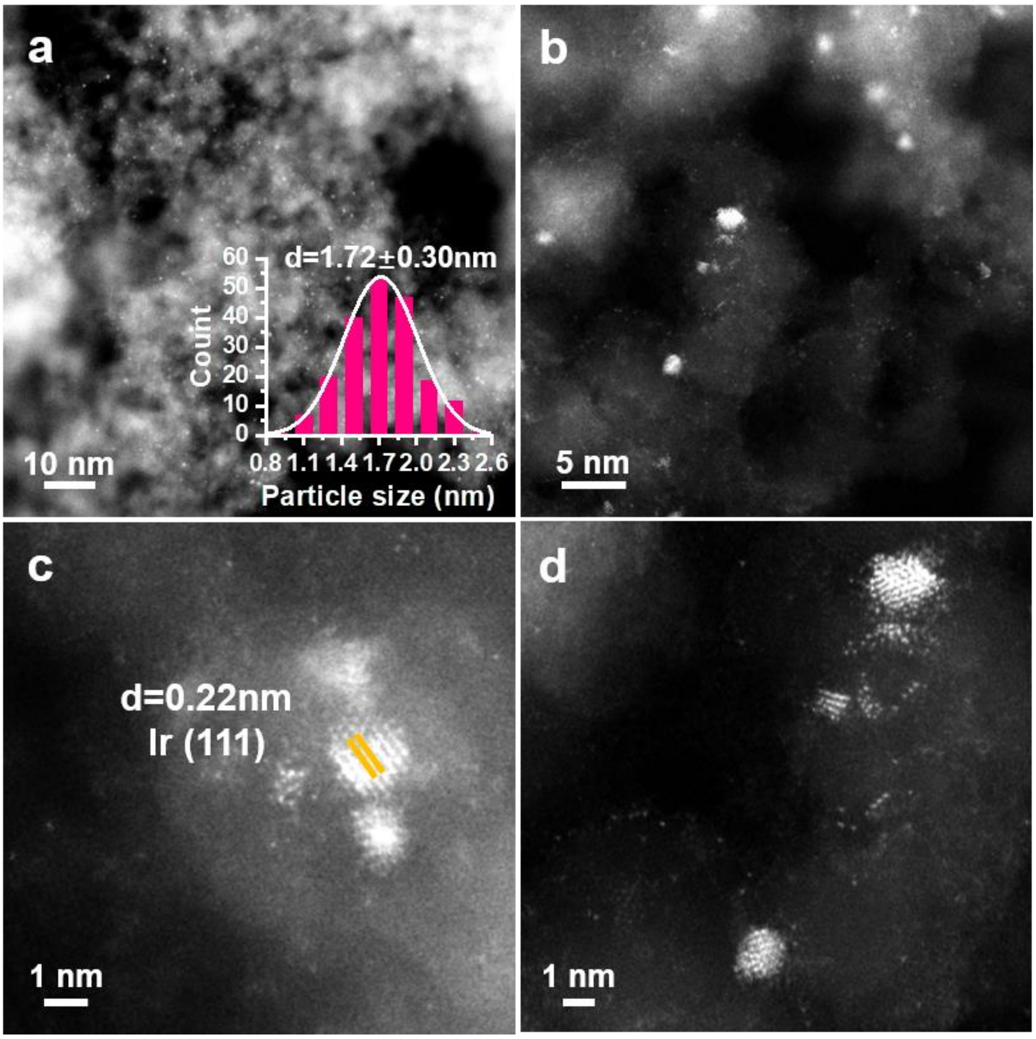

Supplementary Figure 4. HAADF-STEM images of IrNPs/ND@G. (a) The image of IrNPs/ND@G at low magnification. The inset is the particle size distribution of IrNPs/ND@G. (b-d) The image of IrNPs/ND@G at high magnification.

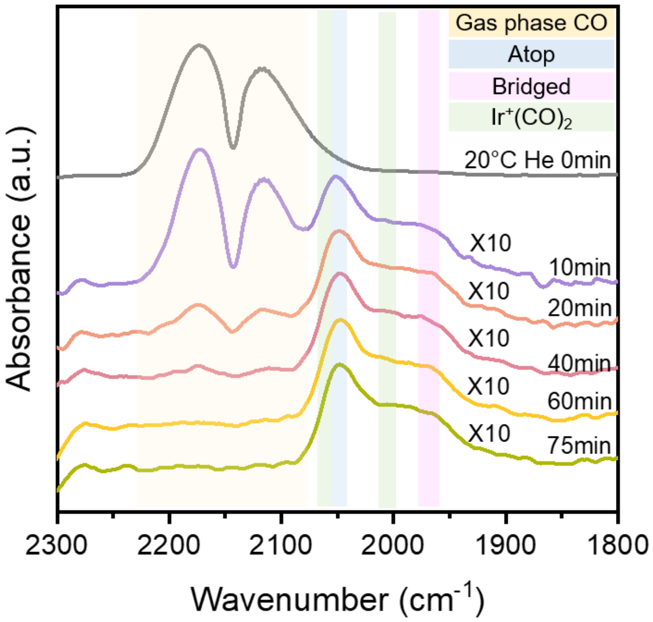

Supplementary Figure 5. *In-situ* CO-DRIFTS of IrNPs/ND@G.

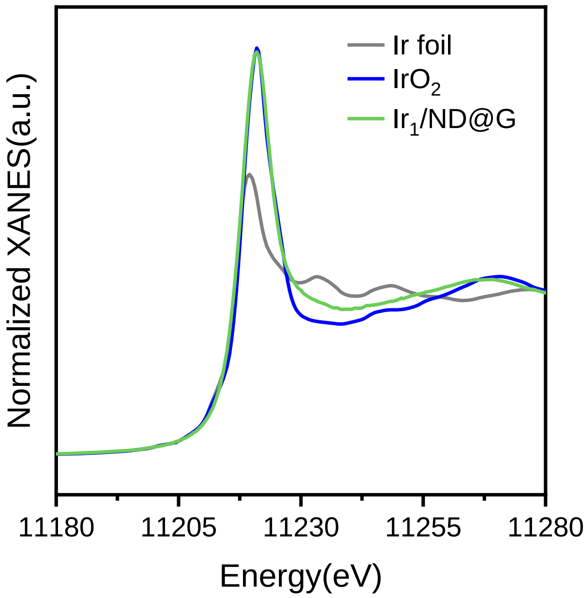

Supplementary Figure 6. Ir L_3 XANES spectra of Ir_1/ND@G, Ir foil and IrO_2.

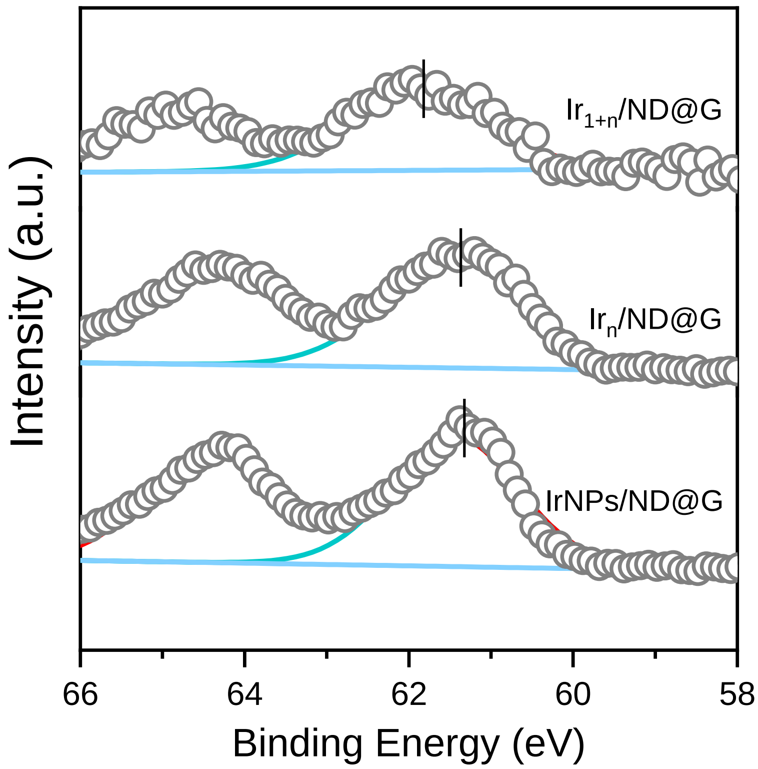

Supplementary Figure 7. Pt 4*f* XPS spectra of Ir_{1+n}/ND@G, Ir_n/ND@G and IrNPs/ND@G.

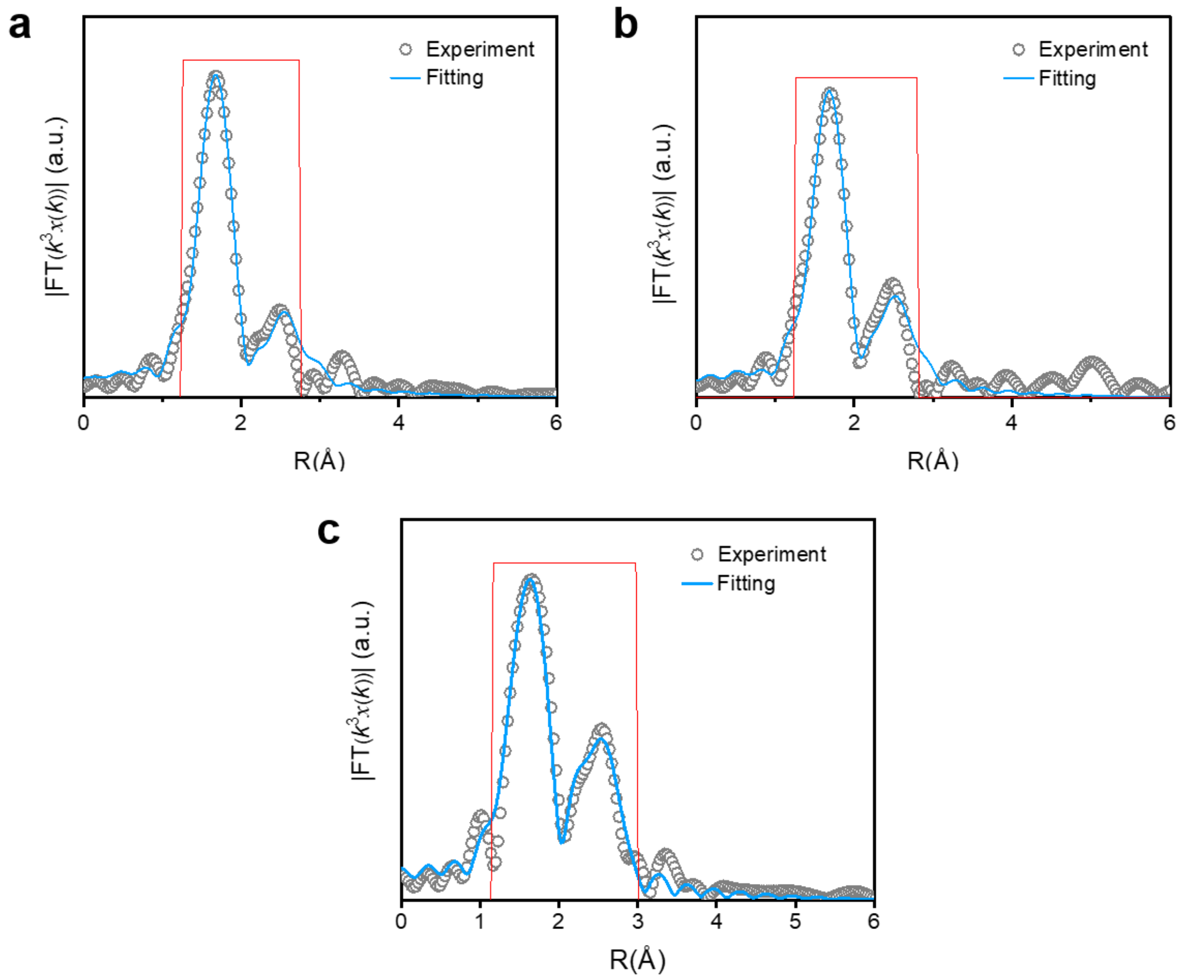

Supplementary Figure 8. EXAFS fitting results of Ir L_3-edge in R space. (a) Ir_{1+n}/ND@G, (b) Ir_n/ND@G and (c) IrNPs/ND@G.

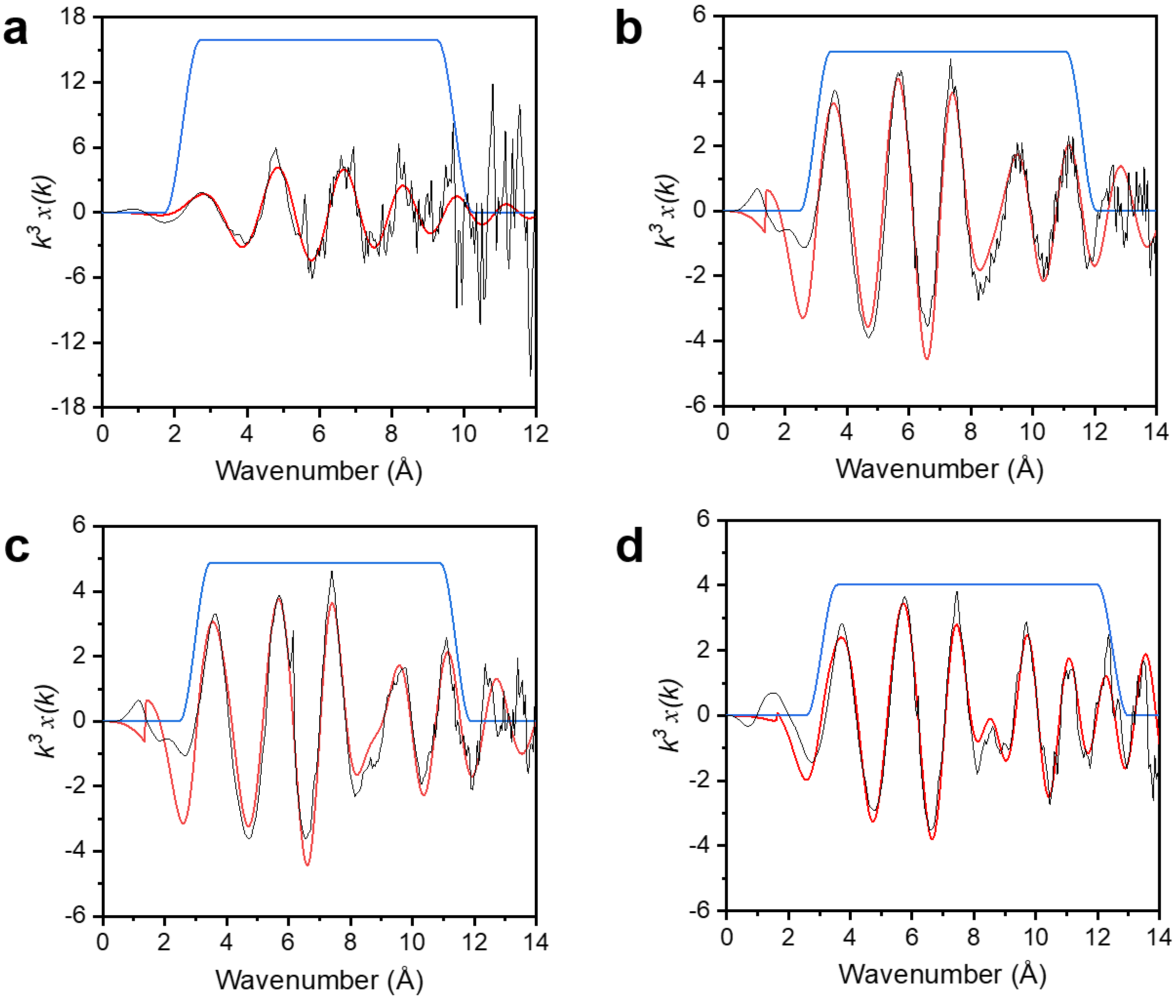

Supplementary Figure 9. EXAFS fitting results of Ir L_3-edge in k^3 space. (a) Ir_1/ND@G, (b) Ir_{1+n}/ND@G, (c) Ir_n/ND@G and (d) IrNPs/ND@G.

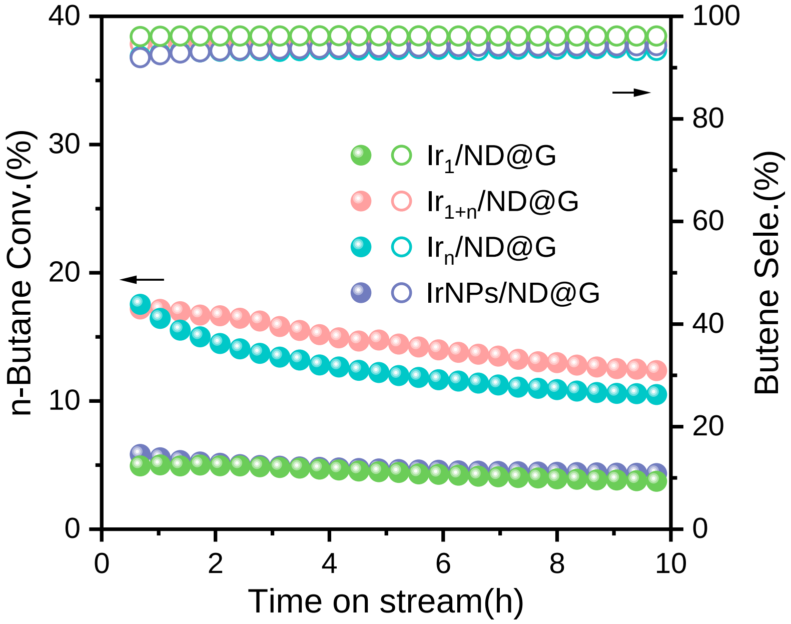

Supplementary Figure 10. The catalytic performance of as-prepared catalysts. n-Butane conversion and butene selectivity by time-on-stream during BDH at 450 ℃, GHSV = 45000 $mL \cdot g_{cat}^{-1} \cdot h^{-1}$, n-$C_4H_{10}$: H_2 = 1:1 with He balance.

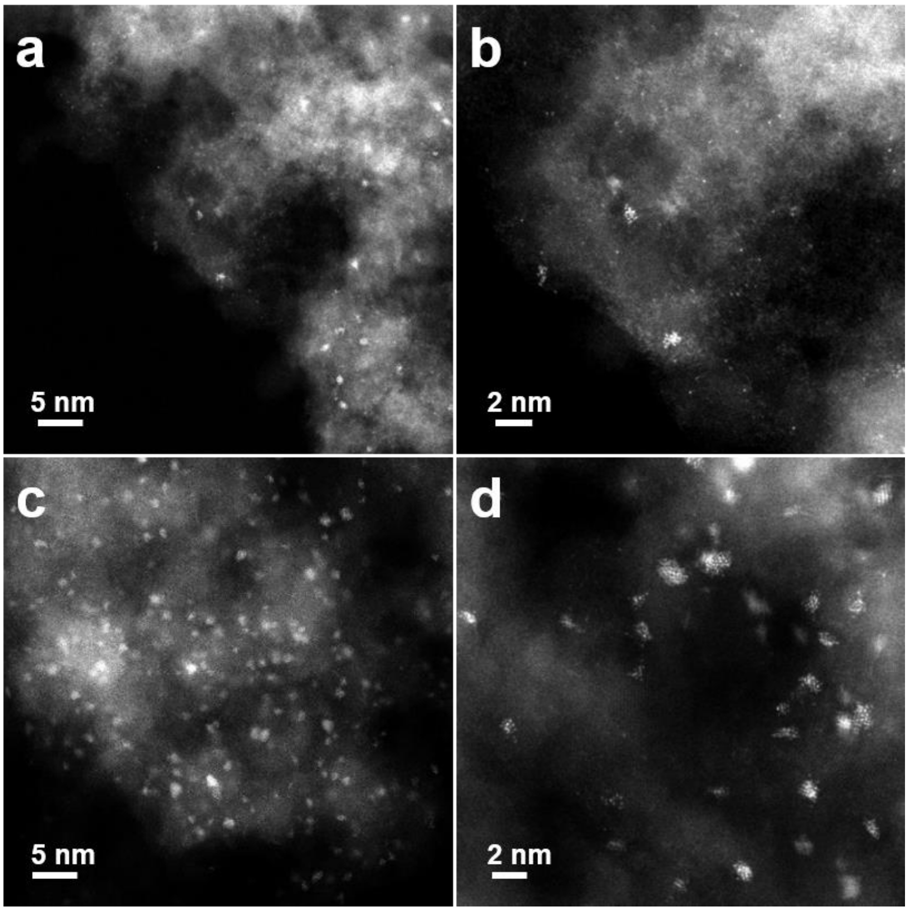

Supplementary Figure 11. Microscopic characterizations of Ir catalysts after 10h BDH. (a) and (b) HAADF-STEM images of Ir_{1+n}/ND@G. (c) and (d) HAADF-STEM images of Ir_n/ND@G.

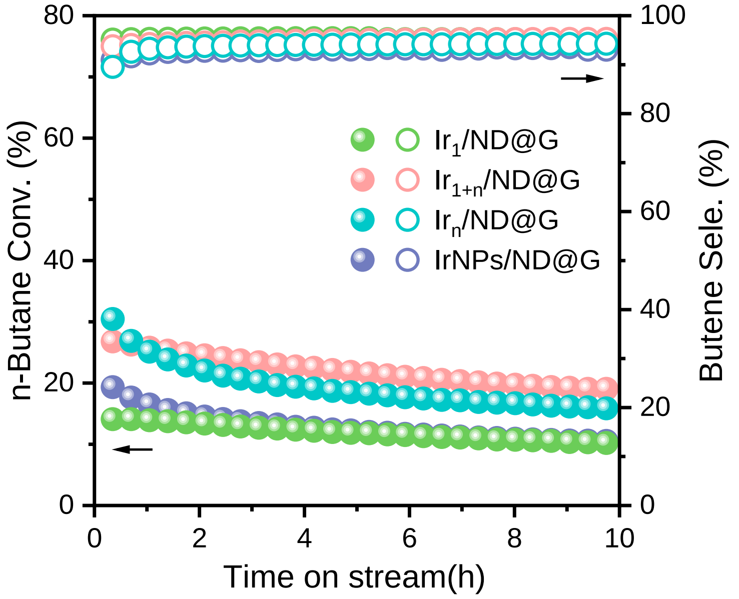

Supplementary Figure 12. The catalytic performance of 50mg as-prepared catalysts. n-Butane conversion and butene selectivity by time-on-stream during BDH at 450 ℃, GHSV = 18000 mL · g_{cat}^{-1} · h^{-1}, n-C_4H_{10} : H_2 = 1:1 with He balance.

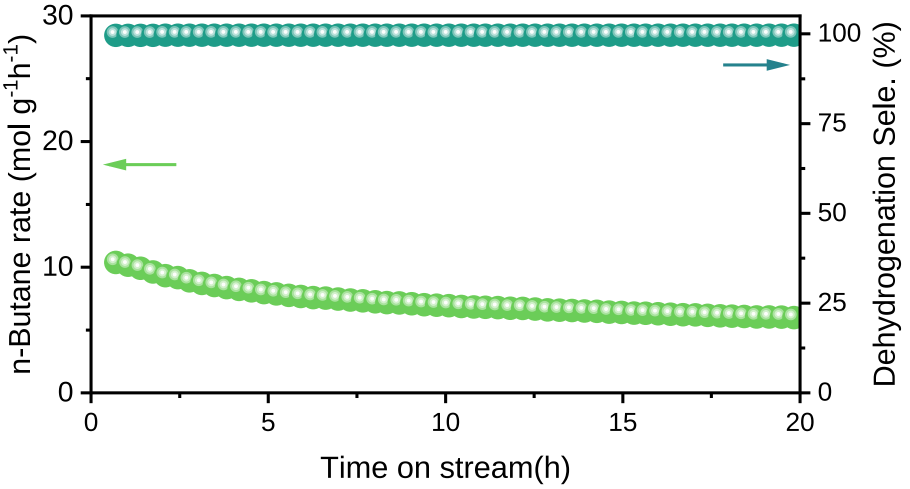

Supplementary Figure 13. Stability test over Ir_1/ND@G at 450 ℃ for 20 hours.

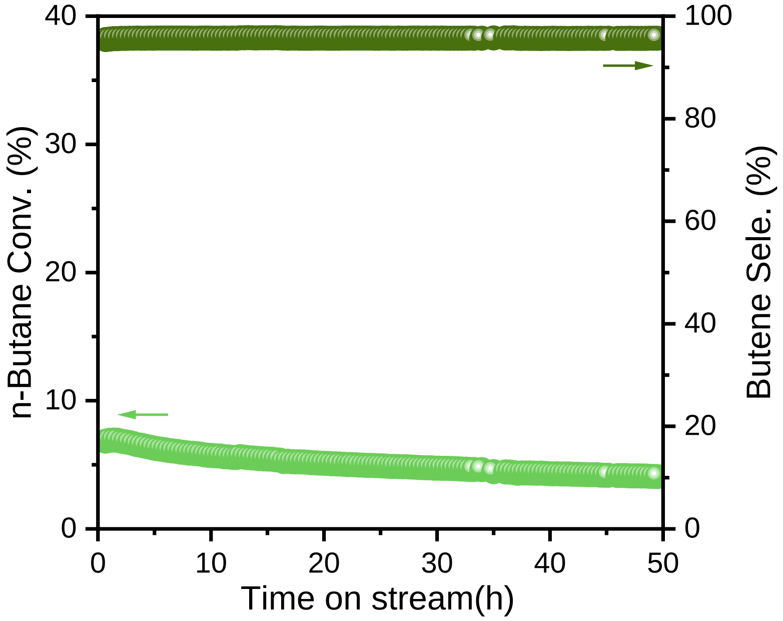

Supplementary Figure 14. Stability test over Ir_1/ND@G at 450 °C for 50h. Reaction condition: GHSV = 30000 mL · g_{cat}^{-1} · h^{-1}, n-C_4H_{10}:H_2 = 1:1 with He balance.

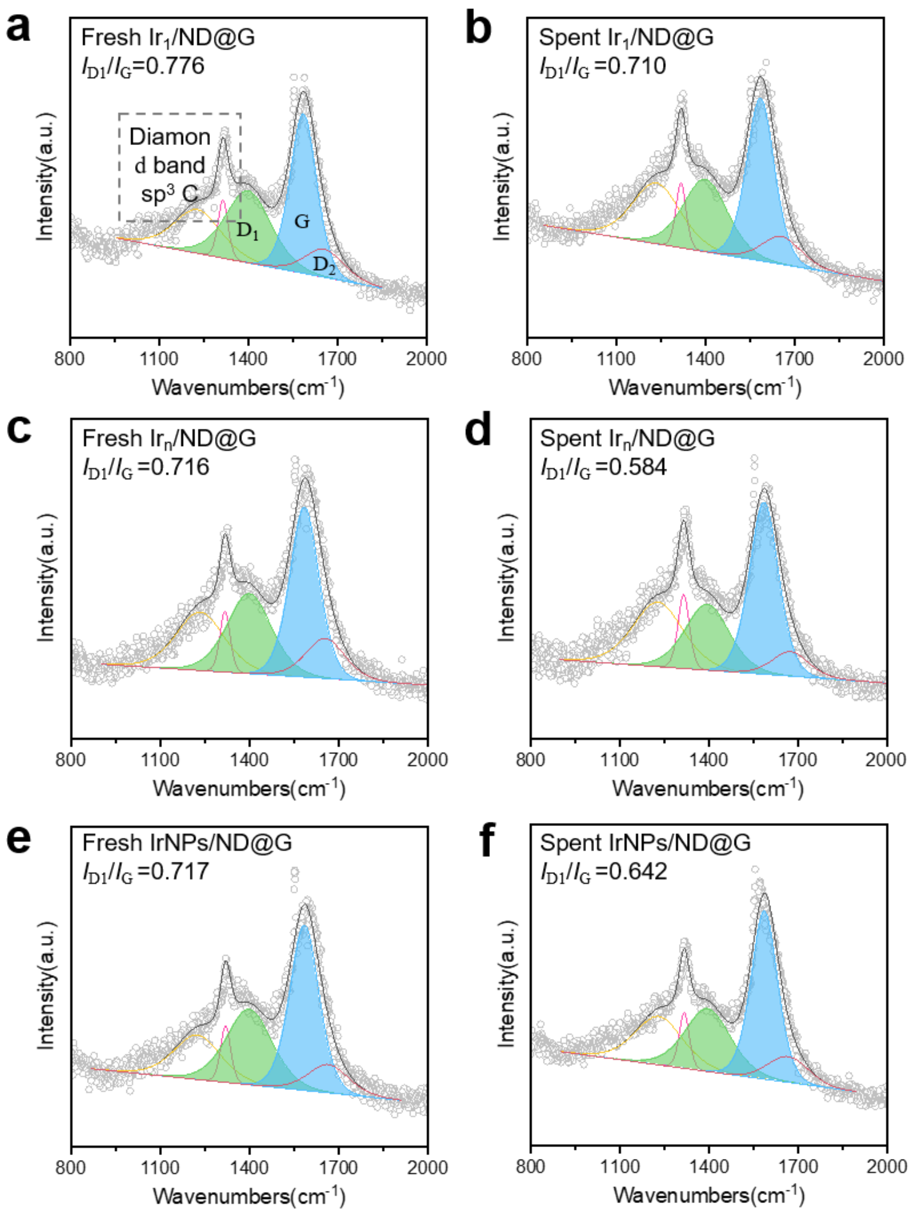

Supplementary Figure 15. Raman spectra for the fresh and spent catalysts after 10-hours BDH. Raman spectra of (a) fresh Ir_1/ND@G, (b) spent Ir_1/ND@G, (c) fresh Ir_n/ND@G, (d) spent Ir_n/ND@G, (e) fresh IrNPs/ND@G and (f) spent IrNPs/ND@G. The peak at approximately 1400 cm^{-1} could be assigned to the disordered graphitic lattice (D1). The peak at approximately 1580 cm^{-1} could be assigned to the well-ordered nanocrystalline graphite (G). I_{D1}/I_G (the integral areas values of D1-band and G-band) are used to qualitatively evaluate the degree of coke formation. Comparing I_{D1}/I_G ratio between fresh and spent catalysts, the lower I_{D1}/I_G ratio on spent catalysts suggests more graphitic coke deposited during BDH.

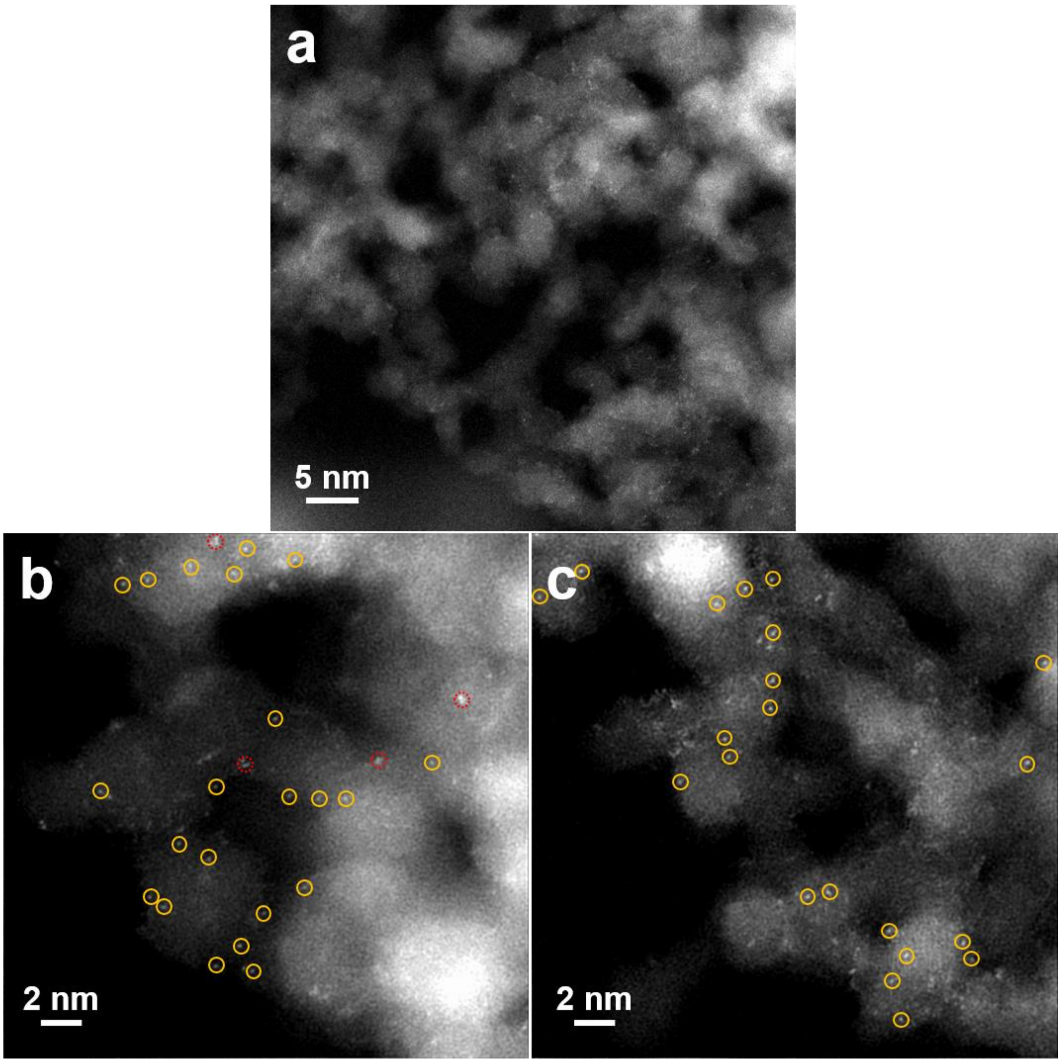

Supplementary Figure 16. HAADF-STEM images of Ir_1/ND@G after 10h BDH. (a) The image of Ir_1/ND@G after 10h BDH at low magnification. (b) and (c) The image of Ir_1/ND@G after 10h BDH at high magnification. The red circles highlighted the small Ir clusters. The yellow circles highlighted single Ir atoms.

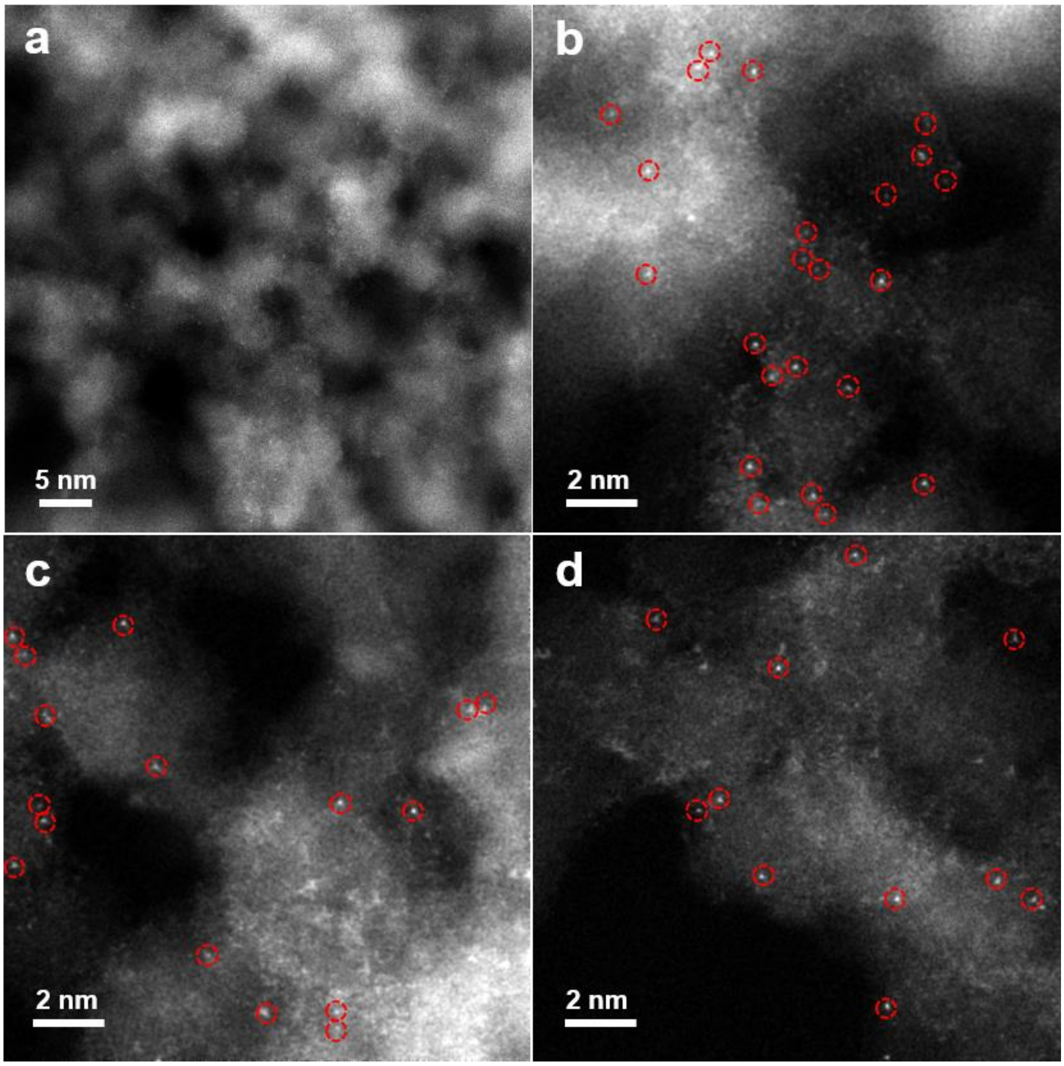

Supplementary Figure 17. HAADF-STEM images of Ir_1/ND@G after 0.5h BDH. (a) The image of Ir_1/ND@G after 0.5h BDH at low magnification. (b-d) The image of Ir_1/ND@G after 0.5h BDH at high magnification. The red circles highlighted single Ir atoms.

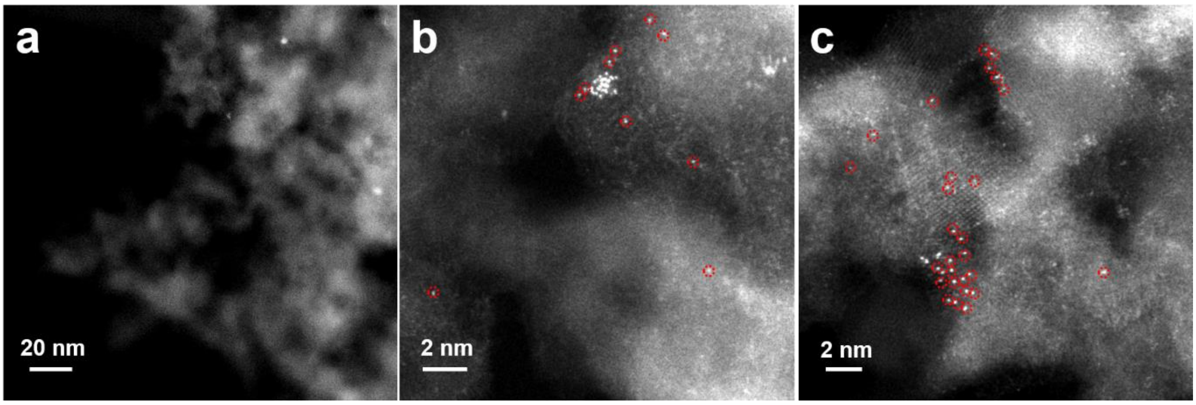

Supplementary Figure 18. HAADF-STEM images of Ir_1/ND@G after 20h BDH. (a) The image of Ir_1/ND@G after 20h BDH at low magnification. (b) and (c) The image of Ir_1/ND@G after 20h BDH at high magnification. The red circles highlighted single Ir atoms.

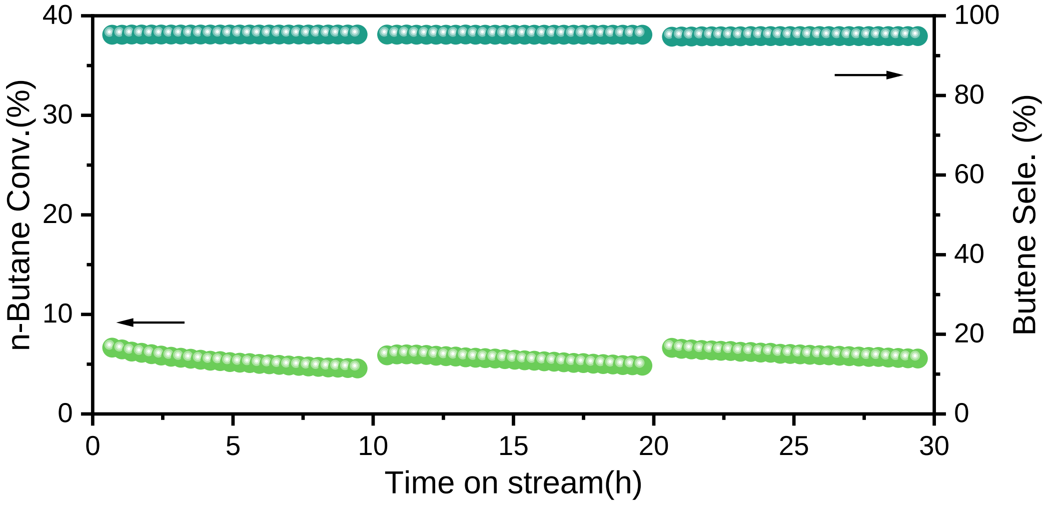

Supplementary Figure 19. The conversion of butane and selectivity of butene over Ir_1/ND@G catalyst after several regeneration cycles. Reaction condition: atmospheric pressure, GHSV = 30000 $mL \cdot g_{cat}^{-1} \cdot h^{-1}$, C_4H_{10}:H_2 = 1:1, He balance, 450 °C.

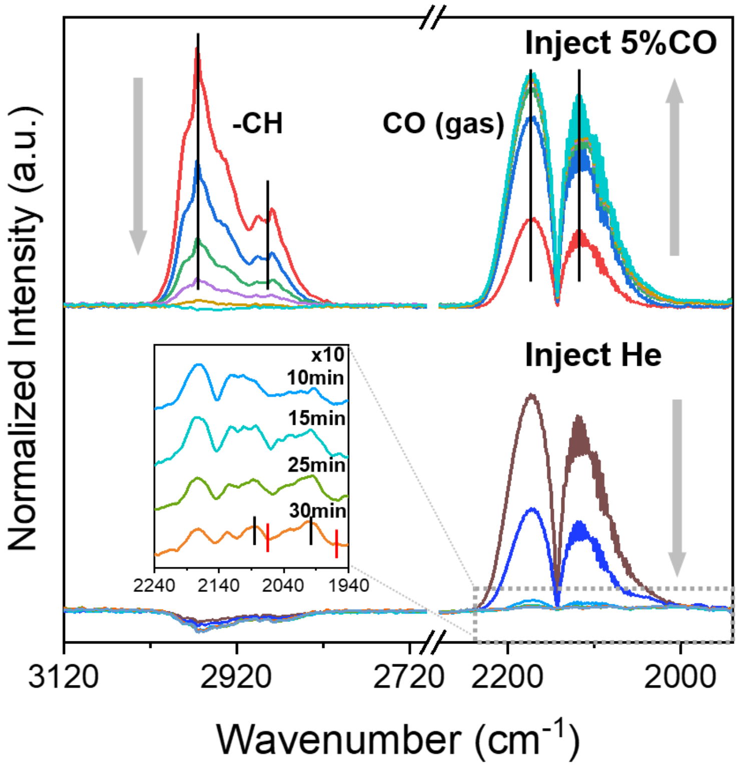

Supplementary Figure 20. The *in-situ* CO-DRIFTS of Ir_1/ND@G under reaction condition for 1h. Reaction conditions: 450 °C, 2%C_4H_{10}, 2%H_2, He balance.

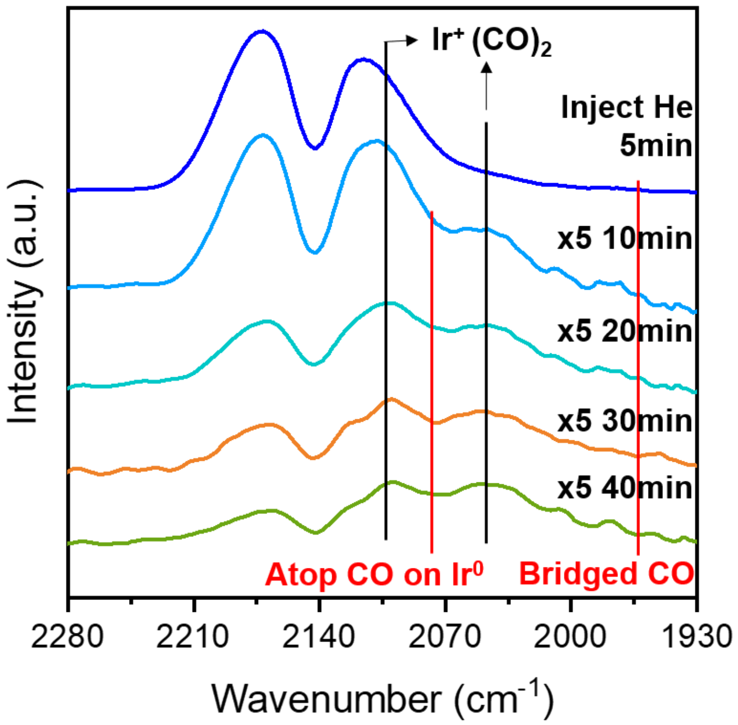

Supplementary Figure 21. The *in-situ* CO-DRIFTS of Ir_1/ND@G under reduction condition for 1h. Reduction conditions: 450 °C, 10%H_2/He.

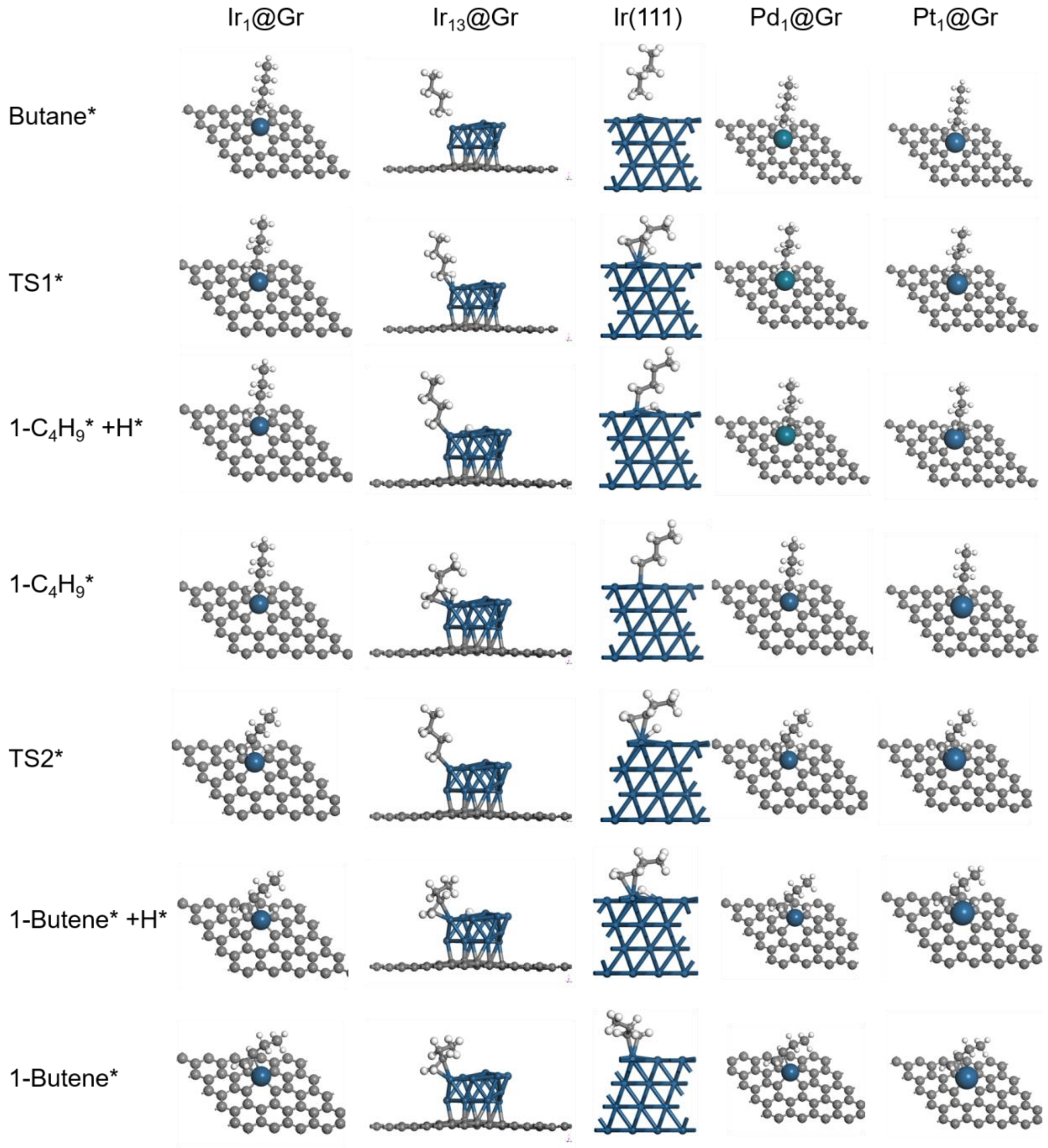

Supplementary Figure 22. The optimized configurations of surface intermediates on Ir_1@Gr, Ir_{13}@Gr, Ir(111), Pd_1@Gr and Pt_1@Gr.

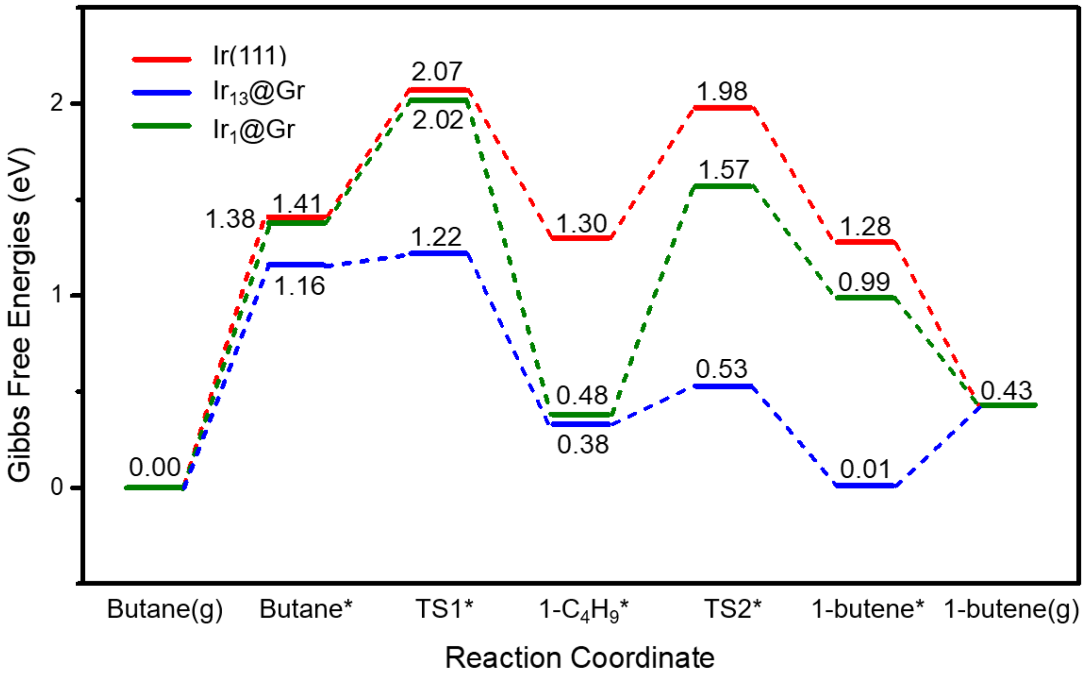

Supplementary Figure 23. Gibbs free energies profiles of BDH on Ir(111), Ir_{13}@Gr and Ir_1@Gr.

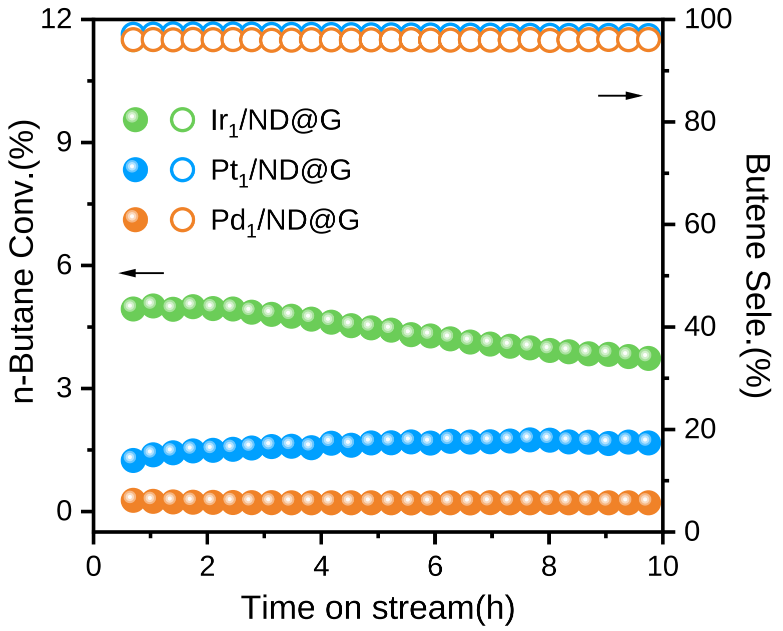

Supplementary Figure 24. n-Butane conversion and butene selectivity of Ir_1/ND@G, Pt_1/ND@G and Pd_1/ND@G.

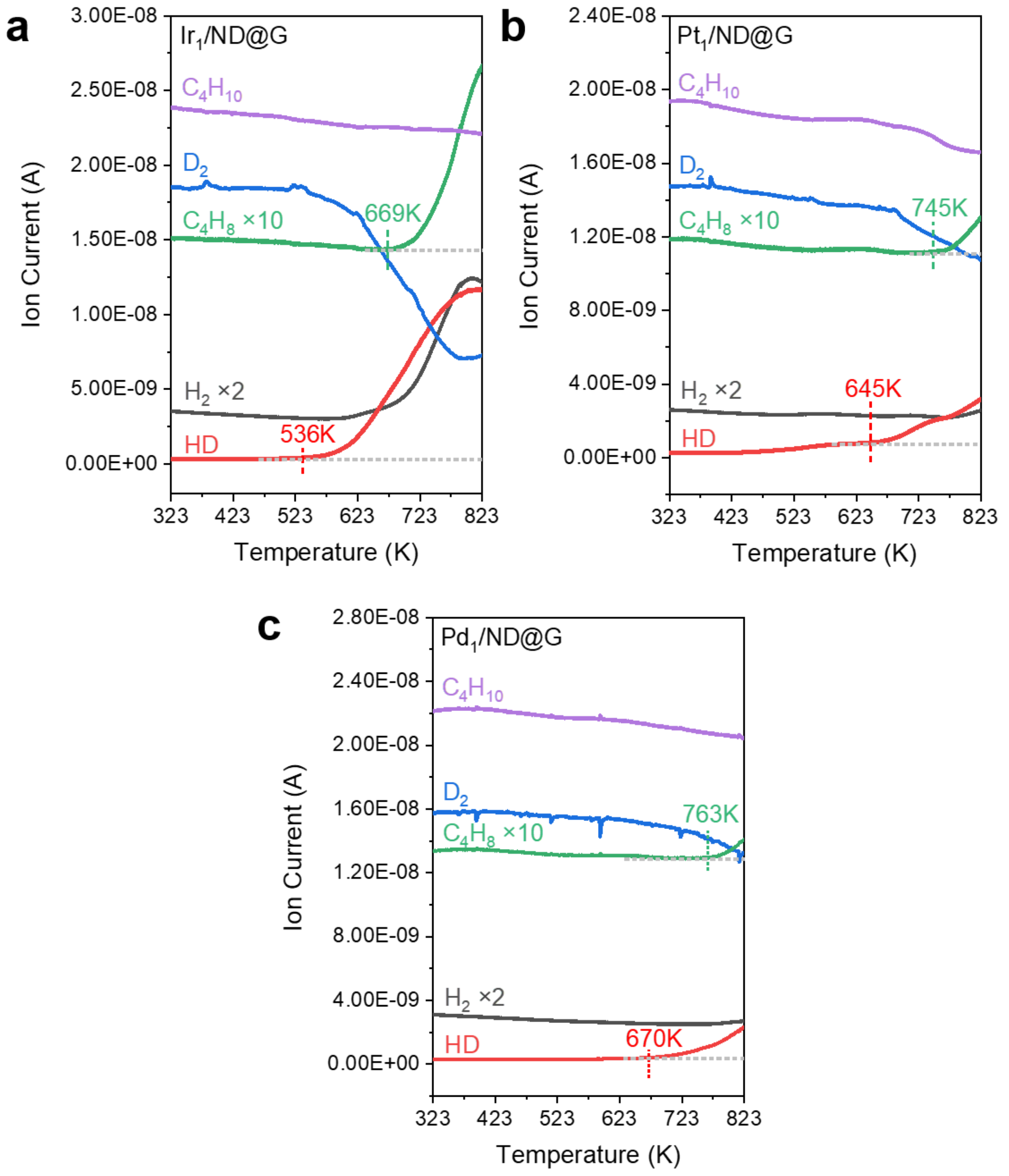

Supplementary Figure 25. TPSR profiles of the mixture of n-butane and D_2 on the different catalysts. (a) Ir_1/ND@G, (b) Pt_1/ND@G and (c) Pd_1/ND@G.

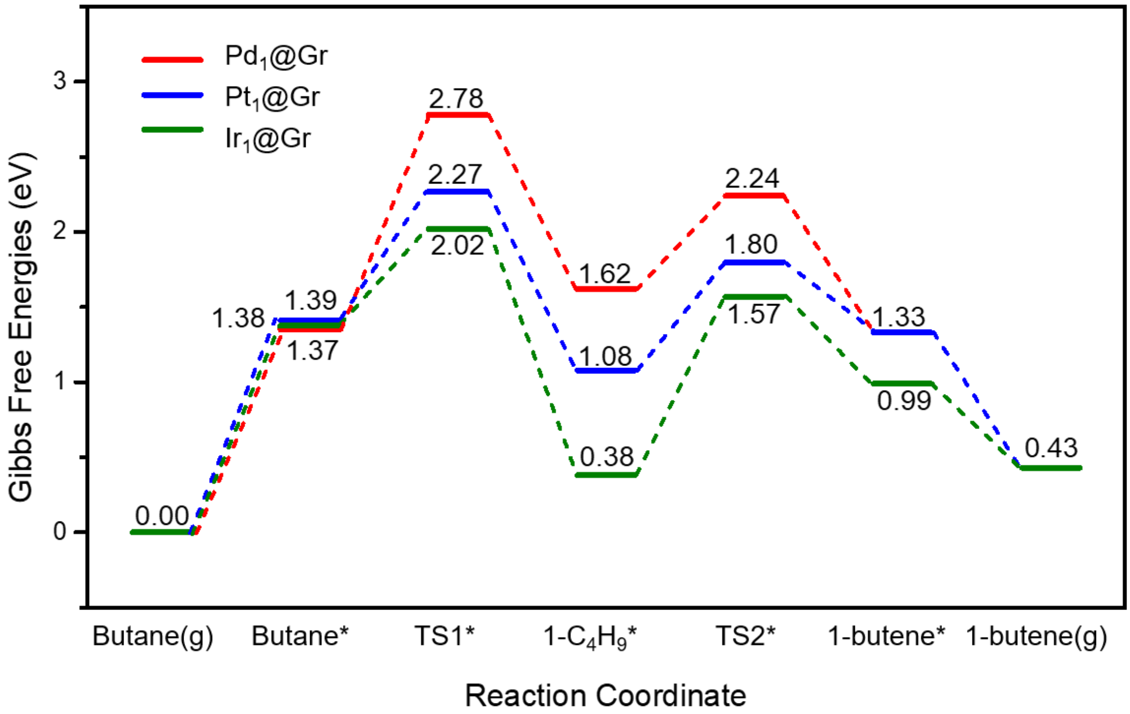

Supplementary Figure 26. Gibbs free energies profiles of BDH on Pd_1@Gr, Pt_1@Gr and Ir_1@Gr.

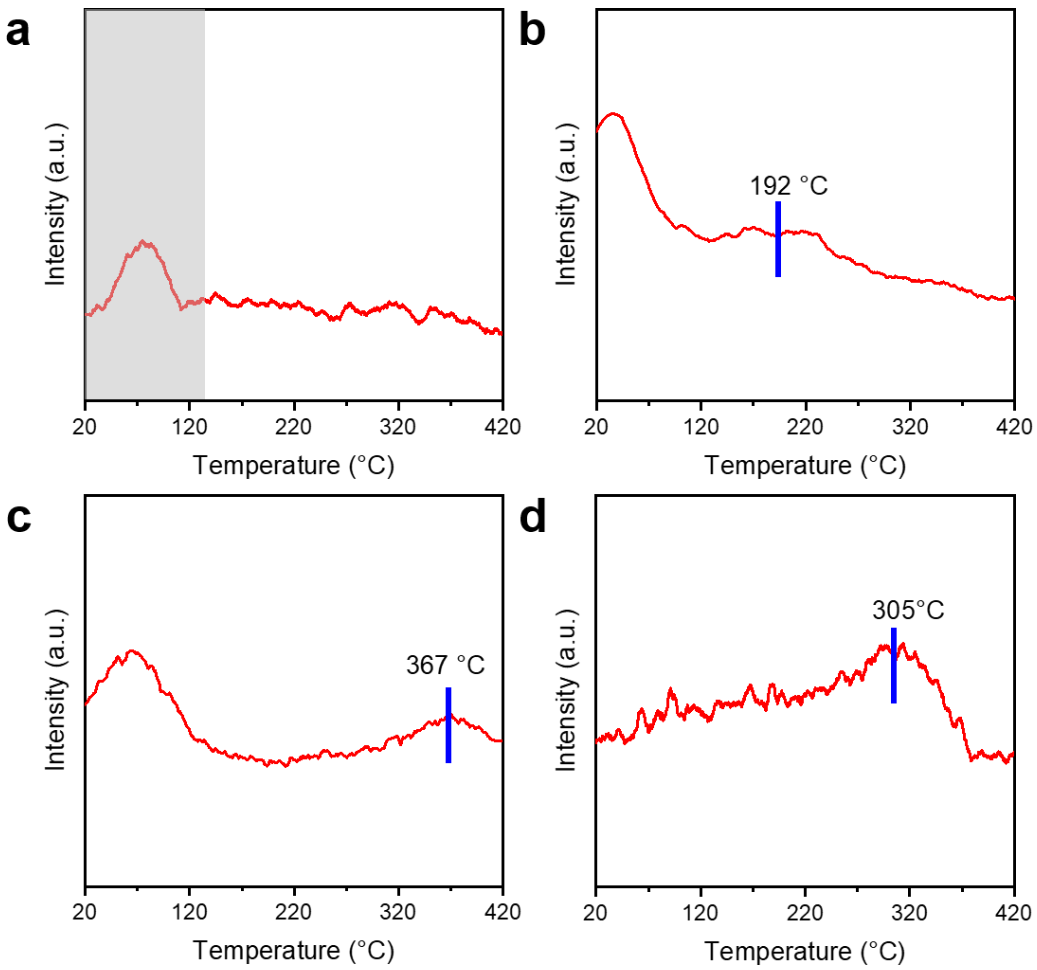

Supplementary Figure 27. C_4H_8-TPD profiles of the different catalysts. (a) ND@G, (b) Ir_1/ND@G, (c) Ir_n/ND@G and (d) Pt_3/ND@G. The peak at 80 ℃ is corresponding to physical absorption on ND@G.

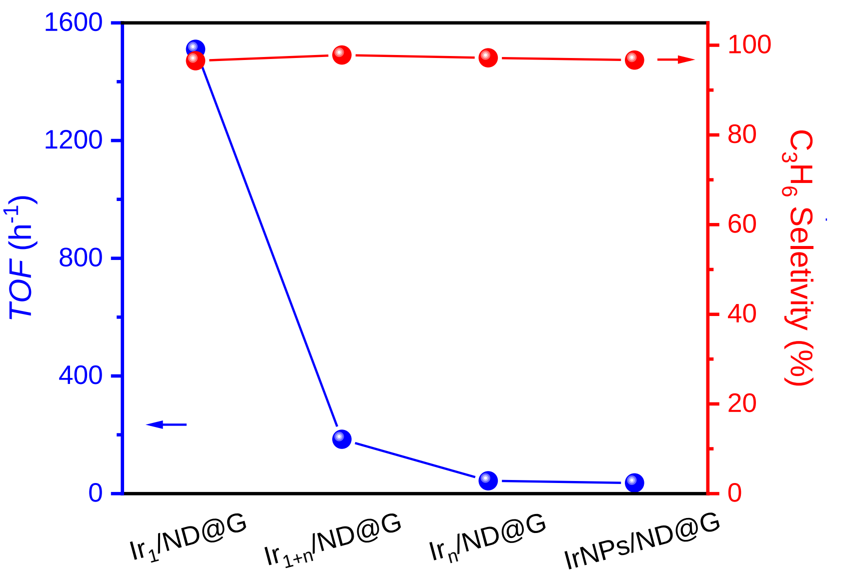

Supplementary Figure 28. TOF and C_3H_6 selectivity over Ir_1/ND@G, Ir_{1+n}/ND@G, Ir_n/ND@G and IrNPs/ND@G.

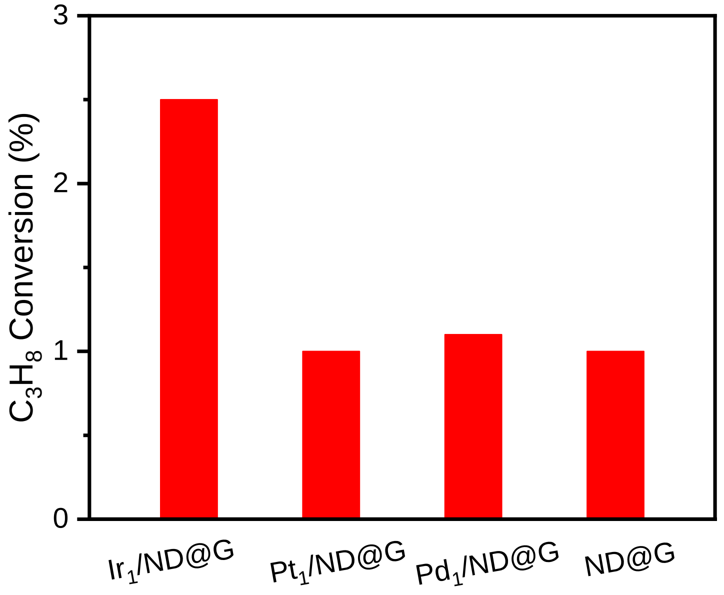

Supplementary Figure 29. C_3H_8 conversion over Ir_1/ND@G, Pt_1/ND@G, Pd_1/ND@G and ND@G.

Supplementary Table 1. Physical structure properties of the catalysts.

Catalysts	Ir loading[a] (wt%)	S_{BET}[b] (m^2/g)	V_{pore}[c] (cm^3/g)	D_{pore}[d] (nm)	Average particle diameter (nm)	Ir dispersion[e] (%)
ND@G	—	350.3	1.4	14.5	—	100
Ir_1/ND@G	0.021	353.4	1.5	13.6	—	100
Ir_{1+n}/NDG	0.43	333.0	1.5	15.3	0.53	100
Ir_n/ND@G	1.3	331.4	1.4	14.9	0.77	100
IrNP/ND@G	1.5	354.9	1.5	14.7	1.72	58.1

[a]determined by ICP-OES; [b]BET method; [c]Volume of N_2 at p/p_0 = 0.98; [d]N_2 desorption; [e]Ir dispersion=1/average particle diameter.

Supplementary Discussion: As shown in **Supplementary Table 1**, not all samples have the dispersion of 100 %. The average particle diameter of clusters in Ir_{1+n}/ND@G or Ir_n/ND@G was less than 1 nm. Calculated by the equation (Ir dispersion=1/average particle diameter), the Ir dispersion was 100 %, indicating Ir species was atomically dispersed and fully exposed on ND@G. For IrNPs/ND@G, the average particle diameter of Ir NPs was 1.72 nm. Calculated by the equation, the Ir dispersion was 58.1 %. The difference in Ir dispersion has been considered during TOF calculations.

Supplementary Table 2. Ir L_3-edge EXAFS fitting results for as-prepared catalysts.

Samples	Shell	R (Å)[a]	C.N.[b]	σ^2 ($Å^2$)[c]	E_0 shift (eV)[d]	R-factor (*10^{-3})
Ir foil	Ir-Ir	2.71	12	0.003	7.9	3.5
IrO_2	Ir-O	1.98	2	0.003	11.3	5.4
Ir_1/ND@G	Ir-C/O	1.98	3.6	0.007	-8.2	8.0
Ir_{1+n}/ND@G	Ir-C/O	2.04	1.4	0.005	8.3	10.5
	Ir-Ir	2.67	2.6	0.010		
Ir_n/ND@G	Ir-C/O	2.03	1.2	0.005	8.2	12.3
	Ir-Ir	2.67	3.5	0.008		
IrNPs/ND@G	Ir-C/O	2.00	3.3	0.007	9.8	18.9
	Ir-Ir	2.69	3.6	0.006		

[a]R is interatomic distance (the bond length between Ir central atoms and surrounding coordination atoms).
[b]C.N. is the coordination number.
[c]σ^2 is Debye-Waller factor (a measure of thermal and static disorder in absorber scatter distances).
[d]E_0 shift is edge energy shift (the difference between the zero kinetic energy value of the sample and that of the theoretical model).

Supplementary Table 3. Catalytic performance of the catalysts for BDH.

Catalysts	Mass (mg)	Time (h)	Conversion (%)	Selectivity (%)			Rate (mol/(g_{Metal}·h))	TOF (s^{-1})
				C_4 olefins	Butene	Others		
Ir_1/ND@G	20	0.35	4.6	99.3	95.6	4.2	8.8	0.48
		9.75	3.7	99.4	96.2	3.8	7.1	
Ir_{1+n}/NDG	20	0.35	17.1	97.4	93.9	6.1	1.6	0.17
		9.75	12.4	98.7	94.9	5.1	1.2	
Ir_n/ND@G	20	0.35	14.7	97.1	91.7	8.3	0.59	0.025
		9.75	9.1	98.4	93.4	6.6	0.32	
IrNPs/ND@G	20	0.35	6.0	96.7	90.7	9.3	0.17	0.014
		9.75	4.3	98.5	94.3	5.7	0.12	
Pt_1/ND@G	50	0.35	1.2	99.5	97.1	3.9	0.20	0.0077
		9.75	1.7	99.5	96.8	3.2	0.26	
Pd_1/ND@G	20	0.35	0.33	100.0	95.8	4.2	0.013	0.0031
		9.75	0.21	100.0	96.1	3.9	0.0085	

Supplementary Table 4. Amount of carbon deposition on the catalysts for 10-hour BDH.

Catalysts	Carbon deposition (wt %)
Ir_1/ND@G	0.10
Ir_n/ND@G	2.08
IrNPs/ND@G	2.12
Pt_3/ND@G	0.64
ND@G	0.06

Supplementary Table 5. Catalytic performance of the catalysts for BDH.

Catalysts	Conversion (%)	Selectivity (%)			K_d (h^{-1})
		C_4 olefins	Butene	Others	
Ir_1/ND@G	13.6/10.2	99.0/99.3	94.8/94.9	4.1/4.3	0.0326
Ir_{1+n}/NDG	26.8/19.0	97.3/98.7	93.7/95.3	4.0/3.4	0.0445
Ir_n/ND@G	30.4/15.9	94.7/98.5	89.5/94.2	5.3/4.2	0.0837
IrNPs/ND@G	19.3/10.5	96.3/98.0	91.0/93.0	5.3/5.0	0.0712

Supplementary Table 6. Summary of the catalytic performance of various supported metal catalysts for BDH.

Catalyst	Mass (g)	Temp. (℃)	Feed	GHVS (mL $g^{-1}h^{-1}$)	TOS (h)	Conv. (%)	Butene Sele. (%)	TOF (s^{-1})	K_d (h^{-1})	Ref
Pd–Cu/Al	0.25	550	C_4H_{10}: H_2: N_2 = 1:3:6	24000	10h	34/17	86.5	0.84	0.0922	1
Pd-Pt/Al						47/39.7	85.5	1.18	0.0297	2
PtSn/Sp-Zn-C	0.2	530	H_2/n-C_4H_{10} = 1.25	5400	2h	28/23	98/98	0.75	0.1319	3
Pt/B/SiO_2	0.05	550	1% n-C_4H_{10}/Ar	2700	25h	1.6/1.5	75/75	0.034	0.0042	4
PtSn/$MgAl_2O_4$	0.2	530	H_2/n-C_4H_{10} = 1.25	5400	2h	31/29	93.8/95	0.71	0.0476	5
PtSnIn(0.5)/γ-A-wc	0.2	530	H_2/n-C_4H_{10} = 1.25	5400	2h	30/28	95	-	0.0486	6
Pt–Sn/SiO_2	0.05	550	20%C_4H_{10}/N_2	24000	13h	33.6/26	99% (total C_4)	9	0.0281	7
PtSn/Al_2O_3	0.03	575	H_2:N_2:n-C_4H_{10}=1:1:1	72,000	10h	37.3/33.8	91.7/91.3	10.2	0.0152	8
Pt/Mg(In)(Al)O	0.005	530	H_2/C_4H_{10} = 2.5	124800	2h	13/7.5	97/97	5.53	0.3057	9
Pt/Sn/Zn/γ-Al_2O_3	0.5	550	C_4H_{10}: N_2=1:1	600	6h	76.0/59.1	62.4/79.1	-	0.1307	10
Pt-TS-1	0.1	500	C_4H_{10}: H_2: Ar = 5: 1: 94	1500	10h	19/20	97 (total C_4)	0.193	-	11
PtMn/SiO2-Reduction	0.005 - 0.055	500	20% C_4H_{10}/N_2	24000	100 h	16/13	99	-	0.002	12
PtMn/SiO2-RWGS						50/18	99	-	0.015	
$Pt_{1.7Sn}$/ND@G	0.05	450	C_4H_{10}:H_2 = 1:1, He balance	18000	10h	35.4/30.9	96.6/96.7	0.12	0.0223	13
Ir_1/ND@G	0.02	450	C_4H_{10}:H_2 = 1:1, He balance	45000	10h	4.6/3.5	95.6/96.2	0.48	0.0241	The work
Ir_n/ND@G						14.7/9.1	91.7/94.3	0.025	0.0749	
IrNPs/ND@G						6.0/4.3	90.7/94.3	0.014	0.041	

Supplementary References

1 Saxena, R. & De, M. Ni/Cu/Ag promoted Pd/Al2O3 catalysts prepared by electroless co-deposition for enhanced butane dehydrogenation. *Mater. Chem. Phys.* **261**, 124236 (2021).

2 Saxena, R. & De, M. Enhanced performance of supported Pd-Pt bimetallic catalysts prepared by modified electroless deposition for butane dehydrogenation. *Appl. Catal. A Gen.* **610**, 117933 (2021).

3 de Miguel, S., Ballarini, A. & Bocanegra, S. New PtSn structured catalysts with ZnAl2O4 thin film for n-butane dehydrogenation reaction. *Appl. Catal. A Gen* **590**, 117315 (2020).

4 Byron, C. *et al.* Role of Boron in Enhancing the Catalytic Performance of Supported Platinum Catalysts for the Nonoxidative Dehydrogenation of n-Butane. *ACS Catal.* **10**, 1500-1510 (2019).

5 de Miguel, S. R., Vilella, I. M. J., Zgolicz, P. & Bocanegra, S. A. Bimetallic catalysts supported on novel spherical MgAl2O4-coated supports for dehydrogenation processes. *Appl. Catal. A Gen* **567**, 36-44 (2018).

6 Bocanegra, S., de Miguel, S., Zgolicz, P. & Ballarini, A. n-butane dehydrogenation on PtSnIn and PtSnGa trimetallic catalysts supported on structured materials prepared by washcoating. *Inorg. Chem. Commun.* **134**, 109033 (2021).

7 Deng, L. *et al.* Elucidating strong metal-support interactions in Pt–Sn/SiO2 catalyst and its consequences for dehydrogenation of lower alkanes. *J Catal.* **365**, 277-291 (2018).

8 Natarajan, P.; Khan, H. A.; Yoon, S.; Jung, K.-D., One-pot synthesis of Pt–Sn bimetallic mesoporous alumina catalysts with worm-like pore structure for n-butane dehydrogenation. *J Ind. Eng. Chem.* **63**, 380-390 (2018).

9 Wu, J., Peng, Z., Sun, P. & Bell, A. T. n-Butane dehydrogenation over Pt/Mg(In)(Al)O. *Appl. Catal. A Gen.* **470**, 208-214 (2014).

10 Seo, H. *et al.* Direct dehydrogenation of n-butane over Pt/Sn/M/gamma-Al2O3 catalysts: Effect of third metal (M) addition. *Catal. Commun.* **47**, 22-27 (2014).

11 Shao, M., Hu, C., Xu, X., Song, Y. & Zhu, Q. Pt/TS-1 catalysts: Effect of the platinum loading method on the dehydrogenation of n-butane. *Appl. Catal. A Gen* **621**, 118194 (2021).

12 Liu, Y. *et al.* Promoting n-Butane Dehydrogenation over PtMn/SiO2 through Structural Evolution Induced by a Reverse Water-Gas Shift Reaction. *ACS Catal.* **12**, 13506-13512 (2022).

13 Zhang, J. *et al.* Tin-Assisted Fully Exposed Platinum Clusters Stabilized on Defect-Rich Graphene for Dehydrogenation Reaction. *ACS Catal.* **9**, 5998-6005 (2019).
